# Impact of Environmental Risk Factors on Mitochondrial Dysfunction, Neuroinflammation, Protein Misfolding, and Oxidative Stress in the Etiopathogenesis of Parkinson’s Disease

**DOI:** 10.3390/ijms231810808

**Published:** 2022-09-16

**Authors:** Minhong Huang, Alejandra Bargues-Carot, Zainab Riaz, Hannah Wickham, Gary Zenitsky, Huajun Jin, Vellareddy Anantharam, Arthi Kanthasamy, Anumantha G. Kanthasamy

**Affiliations:** 1 Department of Biomedical Sciences, Iowa State University, 2062 Veterinary Medicine Building, Ames, IA 50011, USA; 2 Center for Neurological Disease Research, Department of Physiology and Pharmacology, University of Georgia, 325 Riverbend Road, Athens, GA 30602, USA

**Keywords:** environmental factors, pesticides, metals, mitochondrial dysfunction, neuroinflammation, histone modification, protein misfolding, oxidative stress, neurotoxicity, Parkinson’s disease

## Abstract

As a prevalent progressive neurodegenerative disorder, Parkinson’s disease (PD) is characterized by the neuropathological hallmark of the loss of nigrostriatal dopaminergic (DAergic) innervation and the appearance of Lewy bodies with aggregated α-synuclein. Although several familial forms of PD have been reported to be associated with several gene variants, most cases in nature are sporadic, triggered by a complex interplay of genetic and environmental risk factors. Numerous epidemiological studies during the past two decades have shown positive associations between PD and several environmental factors, including exposure to neurotoxic pesticides/herbicides and heavy metals as well as traumatic brain injury. Other environmental factors that have been implicated as potential risk factors for PD include industrial chemicals, wood pulp mills, farming, well-water consumption, and rural residence. In this review, we summarize the environmental toxicology of PD with the focus on the elaboration of chemical toxicity and the underlying pathogenic mechanisms associated with exposure to several neurotoxic chemicals, specifically 1-methyl-4-phenyl-1,2,3,6-tetrahydropyridine (MPTP), rotenone, paraquat (PQ), dichloro-diphenyl-trichloroethane (DDT), dieldrin, manganese (Mn), and vanadium (V). Our overview of the current findings from cellular, animal, and human studies of PD provides information for possible intervention strategies aimed at halting the initiation and exacerbation of environmentally linked PD.

## 1. Introduction

PD is a slowly progressing neurodegenerative disorder that predominantly affects elderly populations. Worldwide, up to 10 million people suffer from PD, and approximately 60,000 more Americans are diagnosed with PD every year. The overall incidence rate of PD is expected to significantly increase over time. According to the US Census Bureau population projections, 680,000 people in the U.S. older than 45 had PD in 2010, whereas in 2020, the number had risen to approximately 930,000, and by 2030, the number is expected to be 1,238,000. Clinically, PD is associated with various motor symptoms, including bradykinesia, resting tremor, and rigidity, and a broad spectrum of non-motor symptoms involving hyposmia, sleep disorders, depression, constipation, early satiety, and excessive sweating [1,2]. As a prevalent movement disorder, this disease dramatically impacts the quality of life of affected patients and imposes a long-term socioeconomic burden on families, the healthcare system, and society. According to a systematic analysis of the global PD burden sponsored by the Bill and Melinda Gates Foundation, its burden is substantially increasing due to a longer life expectancy and the consequent increasing number of elderly people, longer disease duration in individuals, as well as the contribution from environmental factors [3].

PD is recognized pathologically by the progressive loss of DAergic neurons in the substantia nigra pars compacta (SNpc) and aberrant deposition of misfolded proteins (α-synuclein aggregation) in Lewy neurites and Lewy bodies (LB). Compared to DAergic neurons in the SNpc, those in the ventral tegmental area (VTA) are more resistant to degeneration in PD. Although it has been more than 200 years since PD was first medically identified as a neurological syndrome by James Parkinson, the pathogenesis of PD remains unresolved. Previous studies have implicated some disease-related genetic alleles to the onset of PD, such as *SNCA*, *LRRK2* (PARK8) and *VPS35* (PARK17) associated with the autosomal-dominant forms of PD, or *parkin* (PARK-2), *PINK1* (PARK6), *DJ-1* (PARK7), and *ATP13A2* (PARK9) that cause autosomal-recessive PD [4,5]. However, with familial PD only accounting for less than 10% of PD occurrences, most cases are sporadic. Over the past decades, mounting evidence from meta-analyses of mechanistic research and epidemiological studies confirms that the risk for sporadic PD is modulated by environmental factors [6,7,8]. This directs attention to the potential environmental risks, such as traumatic brain injury, cigarette smoking, living in a rural area, well water consumption, farming, agricultural land use, and occupational exposure to metals. A systematic review of extensive multiple PD case studies has also validated that the factors above, particularly pesticides/herbicides, solvents, and metals, are strongly associated with an elevated risk of developing PD in the exposed population [8,9,10,11]. Although the male bias (i.e., 1.4-fold) did not change significantly over the years, more frequent occupational exposures in men might contribute to this gender difference [12].

Several pesticides/herbicides have been implicated in the etiology of PD. Among them, the synthetic meperidine analog MPTP has been widely used to mimic pathophysiological features of PD in multiple organisms, including mice, cats, guinea pigs, and nonhuman primates [13]. PQ is a neurotoxic pesticide that is still widely used in the world. It shares similarities in structure with MPTP and is known to increase oxidative derivatives. Prospective case-control epidemiological studies reveal that PQ exposure increases PD risk two-fold [8]. Similar to PQ, rotenone, a natural chemical produced by leguminous plants native to Southeast Asia and South America, can induce loss of nigral DAergic neurons and behavioral changes in humans. However, unlike PQ, rotenone directly inhibits mitochondrial complex I and results in mitochondria deficits [14]. DAergic neurons are responsible for DA metabolism and are autonomous pacemakers, which places them under intense bioenergetic demand and makes them more vulnerable to rotenone-induced oxidative stress compared to non-DAergic neuron populations [15,16]. The synthetic pesticide DDT has been reported to induce the formation of extracellular vesicles [17]. Additionally, DDT disrupts DA transport by inhibiting the vesicular monoamine transporter (VMAT2) and the plasma membrane DA transporter (DAT) [18]. The organochlorine pesticide dieldrin has been shown to trigger epigenetic modification, perturb proteasomal homeostasis, and activate the apoptotic protein kinase C delta (PKCδ) signaling pathway [19,20]. Some evidence also reveals that the severity of all pesticide neurotoxicity depends on the duration and dosage of the exposure, though high-quality epidemiological proof has been challenging to obtain in the real world as the actual exposure duration is difficult to track and most PD subjects only get diagnosed at the onset of late-stage symptoms [21,22]. In addition to pesticides, several heavy metals have also been associated with PD pathogenesis. Excessive exposure to Mn leads to its accumulation in the human brain and triggers neurotoxicity, even resulting in the development of manganism, a PD-like movement disorder [23]. Another metal pollutant, vanadium (V), which easily crosses the blood-brain barrier (BBB), often occurs with other metals in occupational exposure, particularly Mn. V generates iron-mediated reactive oxygen species (ROS) and therefore induces neurotoxic damage to the brain [24]. Oxidative stress from most of these environmental neurotoxins leads to disruption of calcium (Ca^2^^+^) homeostasis [25,26,27]. Studies using cell culture models have demonstrated that transient increases of intracellular free Ca^2^^+^ ions may induce cytoplasmic aggregates of α-synuclein [26]. Interestingly, exposure to environmental neurotoxins not only impacts motor symptoms but also influences the host gut microbiome [28,29].

In addition to neurotoxic pesticides/herbicides and metals, traumatic brain injury (TBI) has also been linked as a risk factor for several neurodegenerative diseases, but the strongest emerging evidence is associated with the development of PD. Inflammation, metabolic dysregulation, and protein accumulation have been implicated as potential mechanisms through which TBI can initiate or accelerate PD. Alpha-synuclein, amyloid precursor protein (APP), hyper-phosphorylated tau, and TAR DNA-binding protein 43 (TDP-43), which are proteins closely associated with PD, are some of the most frequently reported proteins upregulated following TBI [30].

**Figure 1:**
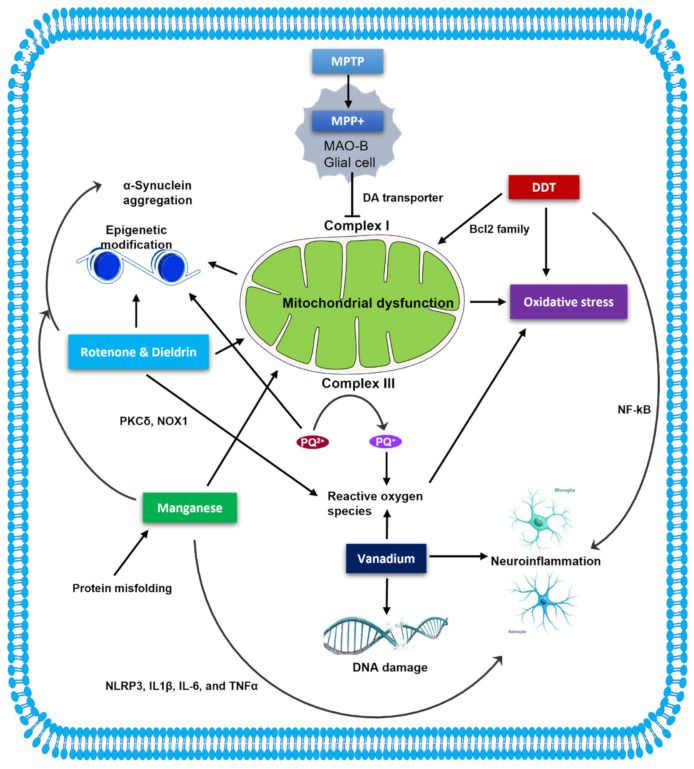
Cellular stress signaling in neurotoxicant-induced neuronal degeneration. Mitochondrial dysfunction is a central driver of PD and plays a significant role in PD pathogenesis. In the brain, MPTP is first metabolized to MPP^+^ by the enzyme MAO-B in glial cells. Upon uptake via DA transporter, MPP^+^ inhibits mitochondrial complex I. Similar to MPTP, rotenone, dieldrin, and PQ inhibit the mitochondrial respiratory chain, induce increased ROS release, and alter epigenetic modifications directly or indirectly via mitonuclear communication. Rotenone and dieldrin can also favor ROS production through PKCδ and NOX1, while PQ induces the generation of superoxide by transforming from PQ^2^^+^ to PQ^+^. Like other pesticides, DDT exposure induces oxidative stress, and it also causes mitochondrial impairment by altering the gene expression of the apoptosis regulator Bcl-2. DTT also induces neuroinflammation through the NFκB pathway. Similarly, excessive Mn increases the levels of inflammatory mediators, such as NLRP3, IL-1β, IL-6, and TNFα. Mn overload likely puts mitochondria under stress and promotes α-synuclein aggregation. V overexposure can not only induce neuroinflammation but also can bring about ROS production and DNA damage.

A critical emerging question is therefore how these diverse neurotoxicants contribute to the pathogenesis of PD. To address it, this review will focus on the chemical neurotoxicity of some key pesticides and metals that may contribute to the disease, specifically MPTP, rotenone, PQ, dieldrin, DDT, Mn and V. We will examine the evidence regarding their molecular and cellular signaling on neurodegeneration from various mechanistic perspectives: mitochondrial dysfunction, neuroinflammation, oxidative stress, histone modification, and protein misfolding/aggregation, as well as a review of our recent findings (Figure 1 and Table 1) [24,31,32,33,34].

## 2. MPTP

Although environmental risk factors for PD have gained considerable attention during the 20th century, definitive proof of the implications of any specific agent as a cause of PD is still inconclusive [124]. The most compelling evidence emerged with discovery of the synthetic heroin analog MPTP in 1982 when several drug users in California developed subacute onset of severe parkinsonism [125]. It is now well established that MPTP induces, in humans, nonhuman primates, and mice, irreversible and severe motor abnormalities replicating all the clinical features of PD, including tremor, rigidity, bradykinesia, and postural instability. Neuropathological data in both primates and mice indicate that MPTP primarily damages the nigrostriatal DAergic pathway in a pattern similar to that seen in PD patients, including the selective loss of DAergic neurons in the SNpc and a significant reduction in striatal DA content [13]. As in PD, the toxin also induces additional neurodegeneration in the locus coeruleus [35,36]. Moreover, reminiscent of PD in humans, an excellent response to levodopa and DA receptor agonists and the development of motor complications after long-term manipulation of levodopa were observed in MPTP-treated primates [126]. Therefore, MPTP administration has been extensively used as a toxicant-induced PD model for studying the disease.

Evidence from epidemiological studies on MPTP showing acute and irreversible Parkinsonism in human and non-human primates demonstrates its inducement of mitochondrial dysfunction and oxidative stress in PD [125,127]. MPTP is a lipophilic molecule that can easily cross the BBB and be metabolized to 1-methyl-4-phenyl-2,3-dihydropyridinium (MPDP) in a reaction catalyzed by the monoamine oxidase B (MAOB) in glial cells. This unstable metabolite is further metabolized to the pyridinium ion (MPP^+^, 1-methyl-4-phenylpyridinium iron), the active toxic compound [36]. MPP^+^ is then selectively taken up by the DA neurons via the dopamine transporter (DAT), where it is concentrated in mitochondria, causes the complex I defect and in turn produces ROS, activating microglia, promoting α-synuclein aggregation, and leading ultimately to cell death [36,37,38,39,40,41]. MPP^+^ causes mitochondrial O_2_^−^ formation, which reacts with endogenous ∙NO to form ONOO^−^. This increases oxidative stress resulting in cyclosporin A (CsA)-sensitive mitochondrial depolarization and Ca^2^^+^ efflux by opening a nonspecific pore in the mitochondrial inner membrane, which leads to oxidant-induced cell death and contributes to the neurotoxicity of MPTP and MPP^+^ [25]. Additionally, MPP^+^ induces Drp1-dependent mitochondrial fission causing mitochondrial fragmentation, which facilitates mitophagy and enhances neuronal death [47,48]. Upon treatment of cells with MPP^+^, the mitochondrial biogenesis-regulating proteins, SIRT1 and PGC1α, are substantially decreased via increased pAMPK [49]. MPP^+^-induced oxidative stress can also activate transient receptor potential melastatin type 2 (TRPM2) channels, which are Ca^2^^+^ permeable non-selective channels highly expressed in SN neurons, leading to Ca^2^^+^ influx that increases calpain activation and subsequent apoptosis [26]. MPP^+^ can also be taken up by the DAergic synaptic vesicles via vesicular monoamine transporter 2 (VMAT2) [128,129,130]. This uptake may cause the cytoplasmic distribution of DA, leading to increased DA-dependent oxidative stress [131]. Many downstream apoptotic events that are responsible for MPTP-mediated degeneration of SNpc neurons have been identified. These include NFκB-dependent transactivation of iNOS [42], up-regulation of JNK [43] and Bax [44], release of cytochrome c and activation of caspase-3 and caspase-9 [45]. In in vivo studies, subacute MPTP exposure increased α-synuclein levels and the number of astrocytes and damaged the BBB without visible motor deficits [46]. However, chronic exposure in adult and aged mice leads to motor defects along with progressive neurodegeneration and induced microglial activation and astrogliosis. Contrary to acute treatments, long-term exposure does not induce mortality [132]. Although some argue that the MPTP-treated monkey PD model lacks DAergic neuronal loss beyond the nigrostriatal system, critical data display a pattern of DAergic denervation as well as olfactory dysfunction resembling PD patients. The comparative evidence suggests the chronically MPTP-treated nonhuman primate model would be a good choice when studying non-motor features [133].

## 3. Rotenone

Rotenone is a botanical pesticide derived from the roots and stems of certain tropical plants. It was widely used as a chemical to control insect pests of crops, animals, and households, and is still used in fisheries management. Since its discovery in the 1930s, rotenone was believed to be relatively harmless to warm-blooded vertebrates, including humans, and was particularly used in organic farming in the form of sprays and other formulations as a broad-spectrum insecticide because of its non-synthetic nature [134,135,136]. Human occupational exposure to rotenone can occur by inhalation during its extraction and preparation, as well as during its formulation and application as a pesticide. Exposure to rotenone can also occur by ingestion of contaminated food and water [135,137]. The practice of mixing different pesticides, coupled with variable concentrations and personal protective measures, makes it difficult to estimate occupational exposure to rotenone [138]. According to the Environmental Protection Agency (EPA) Reregistration Eligibility Decision for Rotenone approved in 2007, rotenone causes high acute toxicity on exposure by oral and inhalation routes (Category I) and low acute toxicity on exposure via the dermal route (Category IV) [139]. However, up until 2006, rotenone was frequently used in the US on food crops [135]. Studies on rotenone persistence in soil and residue in food are sparse. The half-life of rotenone residues under field conditions is reported to be less than 4 days on lettuce, tomatoes [140], cabbage and soil [141], and 4 days on olives, with residue levels higher than maximum residue levels present in olive oil [142]. Soil temperature has been shown to affect rotenone degradation under both field and lab conditions [141,143]. The EPA effectively canceled the registration of rotenone for food uses by 2011 due to the lack of sufficient safety data to establish maximum contaminant levels, and since then only supports rotenone registration for piscicidal purposes as no discernible risk of exposure to toxic rotenone levels is purported from piscicidal use [139,144]. While the EU and Canada also phased out and banned all non-piscicidal sales of rotenone, its current use in global organic farming has been difficult to estimate. Rotenone continues to be exempt from requirements for tolerance [145], so the risk of exposure from imports continues [146].

Rotenone is known to be a potent toxin that inhibits the transfer in complex I of electrons from iron-sulfur clusters to ubiquinone in the mitochondrial respiratory chain, thus blocking oxidative phosphorylation, compromising ATP synthesis [50] and generating ROS [51,52]. Additionally, rotenone has been demonstrated to have microtubule destabilizing activity and to suppress microtubule assembly [53]. Rotenone-induced mitochondrial damage and microtubule dysfunction may lead to apoptosis [51,147] and inhibit cell proliferation [148], respectively. Being a lipophilic compound, rotenone can easily cross biological membranes including the BBB [54]. Mitochondrial complex I inhibition and oxidative stress have been characterized as the pathophysiologic mechanisms underlying PD [149], and epidemiologic evidence suggests a link between chronic rotenone exposure and PD in humans [14,138]. A case-control study by Dhillon et al. [150] found a link between self-reported occupational and environmental exposure to rotenone and the risk of developing PD in an east Texas population. Farmers with occupational exposure to pesticides and their spouses from Iowa and North Carolina were assessed in the Agricultural Health Study, and while effects of other pesticides could not be excluded, associations were found between rotenone use and risk of PD [151]. The Farming and Movement Evaluation Study linked rotenone to PD regardless of protective glove use in a small sample of pesticide applicators [152]. One epidemiological study also reported a higher incidence of PD in farmers with prolonged exposure to pesticides like rotenone in the French agricultural cohort AGRICAN [153].

Experimentally, rotenone has proven to mimic the pathological hallmarks and neurochemical features of PD in various animal models [54,154]. Rotenone PD models show great promise for the investigation of PD-related pathology, neuropathogenesis and gene-environment interactions [54]. Studies show that chronic exposure to rotenone leads to behavioral symptoms of PD in rats whose brains histologically exhibit progressive degeneration of the DAergic neuronal system as well as α-synuclein-rich LB-like inclusions [55,56,57,58]. Similarly, chronic oral administration of rotenone induces DAergic neurodegeneration and motor deficits in C57BL/6 mice [155]. An intrastriatal rotenone rat model showed less TH immunoreactivity in the striatum and SN, indicating a loss of DA neurons [59]. Rotenone treatment of rat embryonic midbrain neuronal cultures selectively induced DAergic neurodegeneration due to microtubule depolymerization, which leads to disruption in vesicular transport and oxidative stress [156]. Studies by Chu et al. [60,61] reported rotenone induces activation of the autophagy protein microtubule-associated-protein-1-light chain-3 (LC3) and redistribution of cardiolipin to the outer mitochondrial membrane, thereby promoting the mitophagy mechanism in both primary cortical and SH-SY5Y neuronal cells. Similarly, it was reported that rotenone induces LC3-positive autophagic vacuole formation, and these vacuoles colocalize with α-synuclein aggregates via oxidative stress and mitochondrial dysfunction both in vitro and in vivo [157]. At high concentrations, rotenone also affects peroxisome morphology and distribution induced by its microtubule destabilizing activity in COS-7 cells, which affects the peroxisome–mitochondria redox relationship and may contribute to PD pathogenesis [53].

Although their neuropathologies are somewhat variable, several studies have established a link between rotenone toxicity and its relevance to PD. It was demonstrated that rotenone decreases phospho-CREB levels and causes degeneration of human DAergic SH-SY5Y cells via the PI3K/Akt/GSK-3β/CREB signaling pathway [62,63]. Rotenone increases intracellular free Ca^2^^+^ ions, which activates calcium/calmodulin-dependent protein kinase II and subsequently induces neuronal apoptosis [70]. Additionally, a calcium channel antagonist prevented rotenone-induced apoptosis in patient-derived DAergic neurons [158]. Rotenone has been shown to promote α-synuclein aggregation and phosphorylation by modulating the calcium/GSK3β signaling pathway in the catecholamine-secreting rat PC12 cells [159]. Silva et al. [160] characterized the biophysical interaction between rotenone and α-synuclein using electron microscopy and Fourier transform infrared spectroscopy to show that rotenone interacts with α-synuclein to accelerate its fibrillation. Ramalingam et al. [64] reported that rotenone treatments induced α-synuclein aggregation in SH-SY5Y cells and mouse midbrain and striatum, as well as reduced TH-positive cell viability.

Rotenone exposure contributes to early neuropathologic mechanisms in PD by altering mitochondrial dynamics. Rotenone-exposed PC12 cells have smaller, fragmented mitochondria and altered levels of proteins involved in mitochondrial fission, fusion and biogenesis [71,72]. In a chronic rotenone exposure model, an early compensatory increase in mitochondrial fusion was later accompanied by detrimental fission [161]. Another study reported functional alteration of mitochondria in rotenone-treated rats and SH-SY5Y cells. The mitochondria appeared abnormal with electron-dense inclusion bodies and both the number of mitochondria and mitobiogenesis markers decreased [162]. Rotenone treatment in SH-SY5Y cells and mouse midbrain and striatum downregulated Parkin expression and upregulated PINK1 expression, which contributes to mitochondrial impairment, oxidative stress and cell death [64]. Chronic rotenone exposure in the SN and striatum of an experimental rat PD model downregulates TH signaling and the cytoprotective proteins Parkin, DJ1 and Hsp70, upregulates Hsp60, and activates caspase-3 and caspase-9 [65]. Rotenone can also promote rapid mitochondrial fragmentation before inducing other cytotoxic cellular changes in primary cortical neurons [163]. Rotenone-induced neurotoxicity is also attributed to NADPH oxidase-derived superoxide release from microglia [66]. In a rotenone rat PD model, pronounced microglial activation occurred prior to DAergic neuronal degeneration [67]. Our studies also show that rotenone treatment significantly impairs mitochondrial respiration in mouse microglia and augments the neuroinflammatory response by promoting microglial PKCδ and NLRP3 inflammasome activation via ROS generation and autophagy dysfunction [68,69].

## 4. Paraquat

PQ is a widely used herbicide in many places around the world. Typical exposure to PQ in humans happens through respiratory inhalation and dermal absorption [164,165,166]. In cases where proper PPE is worn, accidental PQ exposure through respiratory inhalation remains below the threshold limit established by the National Institute for Occupational Safety and Health (NIOSH) [166]. Dermal exposure to PQ is the most concerning. At levels of 5 g/L of the PQ cation in solution, potentially fatal systemic poisoning may occur [165].

Experimental evidence suggests that PQ can dose-dependently generate oxygen-free radicals that are highly damaging to mitochondria, causing oxidative stress, cytochrome *c* release and caspase-9 recruitment, and eventually leading to mitophagy and apoptosis [73,78]. In rat brains, PQ has been observed to use complex III of the electron transport chain to produce H_2_O_2_ [73]. The free radical H_2_O_2_ may also produce O_2_ and HO- [167]. Additional studies in mice show that PQ can induce α-synuclein upregulation and aggregation [79]. In zebrafish embryos, a 24 h exposure to 100 µM of PQ reduces maximal respiration [168]. While no mortality or deformities were visible in the larvae, an up-regulation of certain stress genes and mitochondrial dysfunction did occur, presumably because of increased ROS production [73]. The mRNA levels in two components of the DA signaling pathway, *dat* and *drd3*, were also altered by PQ exposure.

The neural toxicity of PQ is an additional area of interest. In its typical form, PQ is known as PQ^2+^, and is taken up by DAT and OCT3 when in the presence of a reducing agent or NADPH oxidase in microglia [74]. As PQ^2+^, it is not toxic, but as PQ^+^, it increases ROS production and cytotoxicity [74,169]. DAT is the mechanism PQ uses to enter DAergic neurons, while astrocyte entry is made possible through OCT3 [74]. In human SHSY-5Y neuroblastoma cells, PQ causes oxidative stress through ROS production [75]. This increases the rate of caspase-3 activation, leading to apoptosis and DNA fragmentation [75]. Depolarization of the mitochondrial membrane potential also occurs [76]. This study was validated by a similar one in adult rats, which found that rats receiving intraperitoneal injections of PQ thrice weekly showed ~65% DAergic neuron loss within the SN and increased oxidative stress [76]. The SN may be uniquely sensitive to PQ because of its lower percentage of calcium-D28k-containing neurons, which bind Ca^2+^ and can ameliorate some degree of PQ toxicity [170]. Oxidative stress caused by PQ has also been shown to decrease plasma membrane Ca^2^^+^-ATPase activity, leading to Ca^2^^+^ dyshomeostasis and further toxicity [27].

Many people are routinely exposed to PQ. In Thailand, where PQ use is widespread, agricultural field workers, especially pregnant women, had a significantly higher concentration of PQ within their urine and in their children’s meconium than those who did not [171]. Similar findings were reported for mothers who drank community well water, even if they did not live or work on a farm [171]. A shockingly high number of their newborns, 55%, had measurable PQ concentrations [171].

Epidemiological studies on adults have further clarified the potential link between PQ exposure and developing PD later in life. One study focusing on specific gene types in humans and the associated PD risk found that two variations of the *GSTT1* gene had completely different risks with PQ usage. Those with a GSTT1*0 genotype experienced a 7.4-fold greater risk than those with a GSTT1*1 genotype when exposed to PQ [172]. This interaction remained after statistical accommodation for non-PQ pesticide usage [172]. Metabolic genetic variants appear to significantly change an individual’s associated risk of PD due to specific toxicant usage. Despite some epidemiological evidence, the link between PQ usage and PD remains a controversial subject. One common concern in self-reporting studies of this type is that recall memory of specific pesticide use may not always be accurate. Some studies also suggest that to increase PD risk, PQ exposure needs to co-occur with one or more other common toxicants such as Maneb [173].

Due to the accumulating evidence pointing to the involvement of PQ in PD onset, epigenetic modification, especially of histone acetylation, has been under intense investigation. Histone acetylation regulation is responsible for activating differential gene expression, which is crucial throughout life in regulating cellular responses to the environment. Dysregulation of histone acetylation homeostasis can perturb gene expression with detrimental effects. A previous study [77] from our group characterized the disrupted histone acetylation following PQ treatment in N27 DAergic neuronal cells. Exposure to PQ induced acetylation accumulation on the core histone H3 yet kept the acetylation level of histone H4 unchanged in N27 cells. In addition, the PQ insult decreased histone deacetylase (HDAC) activity, particularly HDAC4 and 7. Treatment with a histone acetyltransferase (HAT) inhibitor, anacardic acid, protected against PQ-induced apoptotic cell death by suppressing caspase-3 and PKCδ activity and thus blocked PQ-induced cytotoxicity [77]. These findings suggest dysregulation of epigenetic posttranscriptional modifications of histones as an emerging theme involved in PQ-induced neurotoxicity in DAergic neuronal cells.

## 5. DDT

DDT is an organochlorine pesticide that has been implicated in PD. The popularity of DDT reached its peak around World War II, as a pesticide used to control insect-transmitted diseases such as malaria and typhus (116). DDT-controlled agricultural pests include the European corn borer and the pink bollworm (116). Typical DDT exposure in humans occurs through food consumption. Meat, dairy, poultry, and fish are the primary dietary sources of DDT exposure [174]. Other routes of exposure, such as air and water contamination, are not considered significant [175].

DDT is an effective pesticide and insecticide due to its mode of action. It moves easily into cell membranes using passive diffusion via lipid complexes [176]. Primary neurotoxicity of DDT occurs from CNS excitation enabled by the sustained depolarization of the nerve membrane [80]. This is caused by the combined action of inhibiting both calcium ion transport and the opening of potassium gates as well as by delaying the closing of sodium ion channels [80]. The stereochemistry of DDT may change its neurotoxicity pathway. R-DDT is considered more neurotoxic than S-DDT [81]. In a study looking at PC12 cell exposure to R-DDT, it was found to upregulate p53, NFκB, and caspase 3 [81]. Depending on other toxicants present in the body, the transmembrane potential of the mitochondria can be depressed through exposure to DDT, releasing Ca^2+^ into the cytosol of the cell and triggering various apoptotic factors [84]. These results point toward a cytotoxic pathway that causes apoptosis [81]. In contrast, exposure of PC12 cells to S-DDT caused an increase in SOD, MDA, and HSP70 when compared to the R-form [81]; SOD is an antioxidant commonly upregulated during mitochondrial stress, while MDA is an oxidant. This points to S-DDT inducing oxidative stress rather than activating a cytotoxic pathway. Additional studies suggest that DDT achieves this by inhibiting complexes II and V, encouraging mitochondrial dysfunction and ultimately apoptosis [83,84].

The exact role of DDT and how it relates to PD are still much debated. In vitro studies look promising. One study exposed SK-N-MC cells, which stably produce DA, to DDT [18]. The treated cells showed vesicular VMAT2 and DAT inhibition [18]. VMAT2 and DAT are important for DA transportation, and their inhibition could explain some of the neurotoxic effects of DDT. In mouse embryonic neuronal cells, treatment with DDT reduced the mRNA and protein expression of Bcl-2 and induced apoptosis through caspase-9, caspase-3, and GSK3β [82]. Unfortunately, in vivo studies are much more inconclusive. Exposing mice to DDT at similar or slightly higher levels than the current environmental concentration had no significant effect on stride length, open field activity, or any of the typical markers of neurochemical changes in PD brains such as DAT, VMAT2, TH, α-synuclein aggregation or oxidative stress [18]. A review on pesticide usage and PD heralded DDT not as a cause of PD but as a biomarker of more serious pesticide exposure given its presence in PD brains [18]. While DDT may not directly cause PD, some researchers have implicated DDT in extracellular vesicle formation, which could distribute α-synuclein aggregates [177].

Currently, DDT is still in use in some countries for malaria-bearing mosquitos. DDT binds Na channels by holding them open longer, which enhances the likelihood of action potentials developing, thus creating a condition of hyperexcitability leading to the clinical symptom tremors [178]. DDT mainly metabolizes to two major metabolites DDD and DDE [18]. Increased serum DDE levels were shown to associate with elevated risk for Alzheimer’s disease (AD) [179].

## 6. Dieldrin

Dieldrin was first synthesized in the US in 1946 and was commercially distributed as an insecticide in 1950. It was thereafter extensively used to kill insects of public health importance and on crops, such as corn and cotton, until the USDA canceled all uses of dieldrin in 1970 [180,181]. Soon after that, in 1974, the EPA also suspended the use of dieldrin for agricultural purposes but retained its use in termite control [182], which continued till 1987 when most manufacturers canceled dieldrin registration for use in controlling termites [85,183,184]. Despite not being in use for decades, dieldrin is a persistent pesticide that is ubiquitously distributed in the environment. Due to its lipophilic nature, dieldrin bio-accumulates and bio-magnifies through terrestrial as well as aquatic food chains and can cross the BBB [85,185,186] as this highly toxic insecticide targets the CNS. As an insecticide, dieldrin’s toxicological mechanism of action involves a potent blocking of the GABA receptors, which leads to convulsions and other excitatory effects [85,187]. Dieldrin is acutely toxic and carcinogenic to laboratory animals by inhalation and dermal and oral routes, and the target organ for dieldrin intoxication in these animals is the liver [85,183]. Additionally, dieldrin was found to impair the mitochondrial electron transport chain in the rat liver [87]. In humans, accidental and occupational exposure to dieldrin may occur from ingestion or absorption through the skin. Dieldrin poisoning in humans, for which even acute intoxication can be fatal, is characterized by convulsions and neurological symptoms, such as headaches, dizziness, incoordination, and nausea [85,183].

Increasing evidence from epidemiological as well as in vivo and in vitro studies links dieldrin exposure to DAergic neurodegeneration and PD [85,86]. A few epidemiological studies from as early as the 1990s found a significant correlation between dieldrin accumulation and PD development when comparing postmortem brain samples from PD patients and control cases [85,188,189,190]. Sanchez-Ramos et al. [191] have shown that DAergic neurons are the most sensitive to dieldrin exposure in rat or mouse primary mesencephalic neuronal cultures. More recent studies have also found a stronger association between dieldrin, compared to other persistent organochlorine pesticides, and PD, which supports earlier findings [192,193]. In vivo animal studies also show that dieldrin exposure leads to selective targeting of and neurodegeneration in the DAergic system. Richardson et al. [194] showed that developmental exposure to dieldrin in mice renders DAergic neurons more vulnerable to subsequent exposure to the neurotoxin MPTP, enhancing the MPTP-induced increase in GFAP and α-synuclein levels. These findings are supported by Gezer et al. [195], showing that developmental dieldrin exposure, specifically in male mice, exacerbates α-synuclein preformed fibril-induced striatal DA turnover and motor deficits.

Dieldrin causes neurochemical changes consistent with mitochondrial dysfunction and oxidative stress in the nigrostriatal DA system upon low-level exposure in mice [88], as well as acute exposure in rat DAergic PC12 cells [196,197], which may contribute to apoptotic cell death and PD pathogenesis. We previously observed that dieldrin dose-dependently increases caspase-3 activity, which is followed by PKCδ activation and execution of the caspase-dependent apoptotic pathway in rat N27 DAergic neuronal cells as well as rat brain slices [89]. We further showed the involvement of the pro-apoptotic non-receptor tyrosine kinase, Fyn, in this dieldrin-induced PKCδ-mediated apoptotic cell death pathway [20]. Similarly, Sharma et al. [198] showed that combined exposure to dieldrin and another organochlorine pesticide, lindane, synergistically induced ROS generation and caspase-3/7 activation. Our lab also reported that dieldrin dose-dependently induces ubiquitin-proteasome system dysfunction, as well as exacerbates proteasomal dysfunction in α-synuclein-overexpressing cells, which precedes cell death in DAergic neurons [90]. Dieldrin treatment in N27 DAergic cells similarly results in neurotoxicity and PD pathogenesis by impairing mitochondrial bioenergetics that may be associated with endoplasmic reticulum (ER) stress [199].

In a similar manner to PQ insult, Song et al. showed that dieldrin overexposure stimulates epigenetic histone acetylation modification [19]. However, unlike PQ, exposure to dieldrin in in vitro and in vivo experiments upregulated the acetylation deposition on both histones H3 and H4. Mechanistically, this hyperacetylation is mediated by proteasomal dysfunction and accumulation of HAT [19]. Other studies also show that developmental exposure of C57BL/6 mice to dieldrin increases neuronal susceptibility through DNA methylation at *Nr4a2* and *Lmx1b* genes [200].

## 7. Manganese

Mn is a ubiquitous trace element that is essential for cellular growth, development, and homeostasis. As the 12th most abundant element in the earth’s crust (~0.1%), Mn does not exist in its pure or elemental form naturally but is a component of more than 100 minerals. It presents in trace amounts in all organs of the body. Mn is found in an assortment of food, such as whole grains, nuts, legumes, fruits, tea, leafy vegetables, infant formulas, and some fish and meat. For most people, food is the most common source of Mn exposure [201]. According to human studies, a daily intake range of 1.8 to 2.3 mg Mn/day for adults on Western and vegetarian diets is suggested. In terms of the tolerable upper intake, it is 11 mg Mn/day [202].

Excessive Mn from the environment and industry has been identified as a significant inhaled pollutant. As an environmental risk factor to human health, Mn has been implicated as an etiologic agent in environmentally linked PD and Parkinsonism [7,91,93,103,203,204]. Mn overexposure causes a neurological disorder called manganism, whose clinical manifestation is an extrapyramidal symptom resembling PD and is therefore considered Parkinsonism [7,93,203,205]. The first case of Mn neurotoxicity was from a bleaching powder manufacturer reported by Couper dating back to 1828. The next outbreak occurred in 1912 due to the relatively new technology of chlorine generation using Mn. In 1924, a landmark human autopsy study demonstrated Mn-caused damage in basal ganglia. In 1955, Rodier et al. reported Mn poisoning in Moroccan miners, while in 1932 Beintker et al. showed the first case in welders and Mosheim et al. in battery workers [201,203]. Since then, as the commercial applications for Mn became more widely used, e.g., electric arc welding, battery making, and mineral extraction, the concept of Mn neurotoxicity consequently gained recognition [7,203,206,207,208]. Later, the World Health Organization (WHO, Geneva, Switzerland) and the United States EPA developed guidelines for Mn in drinking water to protect public health. In addition to contaminated drinking water, mining-impacted communities in the U.S.A. also aroused public attention, as other research indicated that infants and children are vulnerable to the harmful effects of Mn dust intoxication [209,210] revealed by a robust and consistent correlation between Mn-containing dust concentration and Mn body burden. School-age children in Brazil with high Mn concentration in their hair have poorer cognitive performance, typically in the verbal domain [211]. Similar findings report a negative association between hair Mn and child IQ scores in East Liverpool, Ohio, USA [212]. For adults, epidemiological studies of 98 cases of Mn-exposed workers indicate the association between welding operations and neurological impairment [213]. The other major sources of Mn exposure in humans include mineral processing, fossil fuel combustion, Mn additive in gasoline (methylcyclopentadienyl manganese tricarbonyl, MMT), metal (alloy, iron, and steel), manufacturing emissions, pesticides (e.g., manganese ethylene-bis-dithiocarbamate, Maneb), fertilizers, Mn violet in paint and cosmetics, dry-cell manufacturing, and a street drug ‘Bazooka’ (a cocaine-based drug contaminated with Mn) [91]. Another psychostimulant drug, methcathinone, also known as ‘ephedrone’ or ‘Russian cocktail,’ presented extrapyramidal abnormalities, alterations in the MRI signal in the basal ganglia, movement disorders, and increased blood Mn in its abusers following multiple intravenous injections for weeks or months, typically due to impurities, including Mn, in this homemade chemical mixture [214,215]. As epidemiological studies have shown, Mn overexposure has a greater neurotoxic impact on the brain than once thought. In general, the relative prevalence of clinical symptoms of Mn toxicity is headache and insomnia (88%), exaggerated tendon reflexes (83%), hyper-myotonia (75%), memory loss (75%), emotional instability (35%), tremor (23%), speech disturbances (6%), and festinating gait (3%) [216]. In an early stage of manganism, some symptoms might be too mild to be recognized.

Neuropathologically, Mn targets the corpus striatum, including putamen, caudate nucleus, and globus pallidus, as an exploratory, neurohistopathological study on prolonged low-level Mn exposure in South African mine workers has shown [93,94]. Manganism’s PD-like neurobehavioral dysfunctions occur in the striatum, while PD impacts the SNpc. Therefore, pathologically unlike PD, whose DA deficiency comes from the loss of DAergic neurons in SNpc, manganism suppresses DA release from the striatum, leading to behavioral deficits similar to PD [92,93]. Changes are not limited only to the basal gangliar region. An increase in olfactory perception, an early neurotoxic indicator, was also seen in a cross-sectional study of 35 male Mn-exposed subjects [217], who also showed significantly higher counts of white blood cells than controls in this study. The altered numbers of leukocytes suggest Mn perturbs the immune system [217]. In clinical manifestations, a meta-analysis of aggregated data from eight studies with 579 Mn-exposed and 433 reference participants found lower performances, short-term memory, and deficits in attention in Mn-exposed individuals. Further details revealed that slow response is the most distinct feature of performances in Mn-intoxicated patients [218]. Overall, human epidemiologic studies of PD patients representing populations from Europe, Asia, and America have well documented the association between Mn overload and increased Mn concentration in the body, PD-like neuropathology, and Parkinsonian syndrome [204,214,219,220,221,222]. However, existing evidence is merely suggestive in linking Mn levels and PD, failing to confirm the certainty that high Mn release significantly elevates PD incidence.

In the body, Mn uptake is affected by three dose-dependent processes involving biliary excretion, intestinal absorption, and intestinal elimination [204]. The efficiency of absorption varies for different Mn salts and exposure routes. For example, Mn chloride is more efficiently absorbed than Mn sulfate or acetate salts. Inhalation is more rapid than ingestion, as inhalation bypasses the control processes of the gastrointestinal tract. Mn is barely absorbed by the skin. Data from animal experiments reveal that following inhalation, small Mn particles are transported in a retrograde direction from the olfactory epithelium directly into the striatum of the midbrain [223]. Through olfactory nerve endings in the striatum, the uptake of Mn may impair brain cells [223]. Interestingly, growing evidence implicates chronic Mn exposure in both occupational and environmental settings in olfactory dysfunction [95,96,97,98,99,100,101,102]. Of note, Mn retention might be greater in infants. Unfortunately, no regulatory maximum of Mn for infant formulas is issued in the U.S.A. [224]. Mn spreads all through the tissues of the body with the highest concentration in the kidneys, liver, pancreas, and adrenals [225]. In contrast, bone and fat have the lowest concentrations [225]. In plasma, most Mn^2^^+^ (~80%) is bound to albumin or α-macroglobulin, whereas merely a small amount of Mn^3^^+^ is bound to transferrin [225]. Within the cell, Mn metabolic homeostasis is regulated by Mn transporters. For membrane transportation, transferrin receptor (TfR), divalent metal transporter1 (DMT1), DAT, ZIP8/ZIP14, calcium channels, choline transporter, citrate transporter, and ceruloplasmin take charge of the influx, while ferroportin, SLC30A10, and NCX are responsible for the efflux [205,225]. For passively transported ion channels, store-operated Ca^2^^+^ channels (SOCC) or voltage-gated Ca^2^^+^ channels (VGCC) escort Mn^2^^+^ across the cell membrane [91]. In terms of intracellular transportation, the Mn level is controlled by TfR and DMT1 in endosomes, PARK9/ATP13A2 in lysosomes, SPCA1, HIP14, SLC30A10 and Ca channels in Golgi, and DMT1, TfR, citrate transporter and Ca transporter in mitochondria [205,225,226].

Mn elimination primarily relies on fecal hepatobiliary excretion and to a lesser extent on urinary excretion [204]. A minimal amount of unabsorbed Mn is excreted in sweat [227]. In the brain, Mn quickly deposits and binds with proteins, forming complexes in structures like the globus pallidus and hippocampus. The half-life of Mn in these regions approximates 5~7 days [204]. In bones, a comparative study estimates that its half-life averages 143 days (range 77–690 days), but in humans, it takes 4.6–41.3 years [204,228].

The cellular mechanisms of Mn neurotoxicity consist of neuroinflammation [23,91,94,229], mitochondrial dysfunction and oxidative stress [34,91,103,104], dysregulated epigenetic modification [105,106,107,108,230,231,232,233,234,235], impaired neurogenesis [110,111,112,236,237,238,239,240,241,242,243,244], and gut dysfunction [28,29]. Neuroinflammation is associated with microglia, the resident immune cells in the brain. Studies on South African miners show higher mean microglia density than non-Mn workers. The longer the miners have worked in Mn mines, the higher the density of microglia in their brains [94]. Microglial activation induced by Mn releases the proinflammatory cytokines IL-1β, IL-6, and TNFα, which are neurotoxic and consequently lead to cellular apoptosis. Evidence from a welding fume study shows that exposure to Mn nanoparticle aggregates remarkably upregulates the inflammation biomarkers IL-6 and IL-8 among Swedish welders. Even though this exposure is below Sweden’s 8-h TWA threshold limit for respirable dust, symptomatic welders showed a tenfold higher level of exhaled breath condensate (EBC) for analysis of leukotriene B4 (LT-B4), compared to people with no symptoms [229]. Inducible nitric oxide synthase (iNOS) produces excessive nitric oxide (NO) during neuroinflammation. Consistent with these results, a human PD study revealed that PD patients and animal models of PD exhibit elevated levels of NO [245]. We found that Mn induces the release of exosomal ASC, which results in NLRP3 inflammasome propagation [23]. The inflammasome is a large macromolecular complex formed by caspase 1, ASC, and the inflammasome component (e.g., NLRPP3, NLRC4, NLRP1, AIM2). The inflammasome plays an essential role in cleaving pro-IL-1β to its mature form IL-1 β or producing IL-18 to enhance and maintain inflammation [246]. Exosomes, as membrane-bound extracellular nanovesicles, function as cargo carriers transporting molecules from one place to another. Serum exosomes and serum from the welders demonstrate both a higher ASC load and more elevated levels of proinflammatory cytokines compared to age-matched subjects [23]. Interestingly, Pajarillo et al. [247] reported that astrocytic transcription factor Yin Yang 1 (YY1) may play a role in Mn-induced neurotoxicity by reducing astrocytic GLAST/GLT-1.

Mitochondrial dysfunction, oxidative stress, neuroinflammation and protein misfolding have been implicated in the pathogenesis of PD. Excessive Mn increases mitochondria-derived ROS production, impairs mitochondrial function, disturbs cellular metabolites, and activates apoptosis-linked cytochrome c release [34,91,103,104]. Mn-induced oxidative stress promotes the accumulation of intra-mitochondrial Ca^2^^+^ by preventing its efflux, thereby inducing mitochondrial dysfunction by disrupting Ca^2^^+^ homeostasis [113]. Huang et al. [248] showed in DAergic SH-SY5Y cells that the mitophagy receptor protein BNIP3 can mediate MnCl_2_-induced mitophagy, leading to neurotoxicity through ROS. Our lab [114] revealed that Mn exposure impairs mitochondrial biogenesis and dysregulates mitochondrial fission/fusion processes in both mouse and human astrocytes, which exacerbates neuroinflammation and Mn-induced DAergic neurotoxicity. We observed that Mn nanoparticles can be effectively internalized, promote upregulation of the Mn transporter protein transferrin, increase ROS release, and activate apoptosis-associated PKCδ in N27 DAergic cells [104]. Further findings reveal that through the PKCδ–PP2A signaling pathway, Mn exposure impairs TH activity in the N27 DAergic neuronal cell line and induces apoptotic cell death [249]. Moreover, in the mitochondrial dysfunction-inherited MitoPark mouse model of PD, a 4-week Mn exposure exacerbated progressive motor deficits, olfactory dysfunction, depletion of striatal DA, nigral TH loss, oxidative damage, and mitochondrial deficits, compared to the untreated MitoPark group [34].

In terms of protein misfolding, Mn induced spatial memory and synaptic plasticity via α-synuclein [250]. Our lab [251] revealed that Mn alters the stability of prion proteins, suggesting its relevance to prion protein misfolding and prion disease pathogenesis.

Similar to pesticides, Mn neurotoxicity can induce abnormal epigenetic modifications. Chronic exposure of human neuroblastoma SH-SY5Y cells to Mn significantly decreases the expression level of the miRNAs miR-7 and miR-433, which reportedly modulate synaptic transmission and apoptosis and target SNCA (the gene that expresses α-synuclein) and FGF-20 (a growth factor) [105]. Mn exposure also induces cellular damage through histone acetylation changes in neuronal PC2 cells [106], while in human neuroblastoma SH-SY5Y cells, Mn alters DNA methylation on *TH*, *PARK2*, and *PINK1* genes that are vitally involved in the onset of Parkinsonism [107]. Human studies of PD patients show that Mn inhalation-exposed subjects have altered DNA methylation (which suppresses transcriptional gene expression, e.g., APC, p16, p53 and RASSF1A) [108,109], histone modifications (e.g., H3K4me2 and H3K9ac on histones from blood leukocytes) [231], miRNA content (e.g., miR-222, miR-21) [232,233], and α-synuclein aggregation-associated miRNA exosome cargo [233]. Interestingly, the levels of DNA methylation in healthy subjects from a mining district in Antofagasta, Chile, are higher than in Santiago, a city having little association with mining [234]. A clinical study in older men added novel evidence to the findings that, due to the close correlation between DNA hypermethylation and toxicity of Mn overexposure, DNA methylation-based measures could be a useful predictor to identify subjects at risk of Mn toxicity-induced disease [235].

In addition to epigenetic dysregulation, Mn overload can impair adult neurogenesis. Neurogenesis in the adult brain functions in cell proliferation, enhanced cell survival, migration to target regions, and differentiation to new neurons [240]. These functions occur in the dentate gyrus (DG) of the hippocampus and the subventricular zone (SVZ) [238,240]. In the neurogenic niche of the SVZ, neural progenitor cells proliferate and migrate through the rostral migratory stream (RMS) into the olfactory bulb (OB) to supply newly generated neurons for neural repair and functional integrity [238,240]. Severe hippocampal atrophy and impaired hippocampal adult neurogenesis have been shown along with motor and non-motor (e.g., depression) symptoms in transgenic animal models and human postmortem brains of PD [110,111,112]. Since airborne Mn is actively transported to the brain via the olfactory tract, the resulting impaired olfactory function serves as a predictive sign of Mn-induced Parkinsonism [244]. Maternal Mn exposure leads to the sustained disruption of hippocampal neurogenesis in the offspring of animal models [108,252]. This malfunction of developmental neurogenesis is mediated by aberrant epigenetic gene regulation through hypermethylation [242]. Interestingly, although Mn overexposure reduces the overall adult neurogenesis in the OB, this exposure initially enhances cell proliferation in the SVZ [237]. Further studies indicate that Mn alters SVZ and RMS neurogenesis by disturbing divalent metal transporter-1 (DMT1) and cellular Cu regulation [236].

Accumulating evidence reveals that the gut communicates with the brain to form a bidirectional signaling axis between the gastrointestinal tract (GIT) and the central nervous system (CNS) through spinal afferents and the vagus nerve, and an abnormal gut microbiome disturbed by environmental factors can be an indicator for early PD symptoms [253,254]. To support this claim, Ghaisas et al. showed that Mn exposure to mice via oral gavage modified gut physiology and altered its metabolic profile [29].

Despite over 3000 publications being listed by PubMed in the past decade related to Mn toxicity research, no protective strategy is available to date. One remedial option is the immediate removal of the exposure source [204]. When comparing improvements in clinical symptoms, levodopa treatment achieved a much poorer response in Mn-poisoned subjects than in idiopathic PD patients, presumably because of the latter’s relatively intact nigrostriatal pathway [255]. A treatment chelation therapy involving EDTA can elevate the toxicant’s excretion in urine and reduce Mn body load [103,205]. However, its efficacy in ameliorating neurological symptoms was under question [206]. Promisingly, several clinical cases reveal the potential efficacy of p-aminosalicylic acid as a treatment [256,257,258]. In another case, a small dose of clonazepam reportedly showed partial success [206]. Unfortunately, currently available treatments for Mn overload are far from satisfactory. To address this shortcoming, recently some scientists turned to mitochondria for exploring new options. It is reported that the amino acid taurine is enriched in the human brain as it is essential in regulating mitochondrial function. An in vitro study indicated taurine protects mitochondria against Mn-induced cytotoxicity [259]. In a Mn-intoxicated in vivo mouse model, taurine mitigated locomotor deficits and oxidative stress, and improved indices of mitochondrial functionality and impaired spatial cognitive ability [260,261]. With respect to interventions at the genetic level, accumulating evidence demonstrates that Mn neurotoxicity induces α-synuclein aggregation and subsequently activates the pathophysiology of PD [262]. By alleviating α-synuclein aggregation, scientists discovered that PARK9 (also known as ATP13A2) protects DAergic neuronal cells from Mn neurotoxicity [263]. We also identified an interesting physiological function of normal α-synuclein that can protect against a neurotoxic challenge during the early stages of Mn exposure in N27 cells stably expressing α-synuclein [264].

## 8. Vanadium

Vanadium (V; atomic number 23), is a ubiquitous transition metal present in most living organisms [265]. As the 22nd most abundant crustal element on Earth [266] and the 2nd most abundant transition element in seawater [267], V is found in 65 different minerals [266]. Its oxidation states range from valences −1 to +5, with +3, +4, and +5 being the most common [268]. V naturally occurs as two isotopes, ^50^V (0.24%) and ^51^V (99.76%), the former having a radioactive half-life of more than 3.9 × 10^17^ years. V is a major trace element in fossil fuels, and the processing and combustion of these materials result in a significant emission of V oxides (V_2_O_4_ and V_2_O_5_) into the atmosphere [266,269]. Other industrial activities that add to the anthropogenic emission of V include the production of ferrovanadium, leachates, and effluents from mining and milling, among others [266]. Atmospheric V occurs in the form of V oxides, about two-thirds of which originate from anthropogenic sources, while the remaining one-third can be traced back to continental dust, marine aerosols, and, to a lesser extent, volcanism [268]. Although exposure to the trace amounts of V emissions in the atmosphere can occur through inhalation, its bioaccumulation in the food chain becomes magnified through its deposition in the soil, groundwater, and vegetation, including crops [270].

This metal is widely used in the manufacture of heat-resistant alloy and glass, pesticides, plastics, semiconductors, photographic developers, coloring agents, sulfuric acid, as well as in petroleum and coal refineries [271,272]. V-reinforced high-strength low-alloy (HSLA) steel has been heavily used in industrial applications such as manufacturing aircraft, tanks, warships, and munitions because of its high structural strength and corrosion resistance despite V being one of the lightest metals [273]. Vanadium use has increased in the manufacturing of high-capacity batteries for energy storage and Li-Fe batteries in electric cars. In addition, V compounds have been investigated for their use in humans as therapeutics to treat diabetes mellitus [274] as well as in the treatment of syphilis, malnutrition, anemia, tuberculosis [275,276], and cancer [277].

Like many essential elements, trace amounts (0.05 μM) of V can be therapeutic and essential to health, yet toxic in excess (>10 μM). In general, V toxicity is low and studies on animals show that the toxic effects of V compounds depend on distinct factors, including V’s physicochemical state, dose, route of administration, and duration of exposure. V’s toxicity rises as its valence increases, peaking in pentavalent compounds. Toxicity is lowest following ingestion, as the GIT absorption of V compounds is poor, intermediate when inhaled, and greatest when administered parenterally [268].

Dietary V is the primary source of exposure for humans with a mean dietary intake of V estimated to be 20 mg/day [278]. Most foods contain <1 ng/g V [268], but this can be quite variable since high amounts of V can be found in black pepper, tea leaves, cocoa powder, and certain mushrooms (165). In addition to foods, drinking water alone can contain from 0.2 to 100 mg/L. The human body contains roughly 100 μg of V [279] with tissue levels accumulating to around 0.3 mg kg^−1^ in bones, liver, kidneys [266,278], and testicles [280,281]. V level in blood plasma is around 200 nM [266], and V is mainly transported via a transferrin-dependent pathway [282].

The extensive production of industrial V-containing dust and fumes during the processing and refining of V ores and sludge, the manufacture of V-containing products, the combustion of V-rich fuels, and the handling of chemical catalysts is the major route of both acute and chronic occupational exposure. Therefore, occupational exposure during V-allied industrial processes and fossil fuel combustion represents major sources of toxicity and likely predisposing factors in the etiopathogenesis of neurodegenerative disease [283].

Neurotoxicological studies in rodent models show that inhalation of V_2_O_5_ damages nigrostriatal DAergic systems [115], the hippocampus [116], and the ependymal epithelium, which opens the CNS to chemical insults normally prevented by the BBB [117]. We have also demonstrated in a rodent model that intranasal V_2_O_5_ exposure reduces tissue volume and DAergic neurotransmission in the OB [118]. Intraperitoneal (i.p.) NaVO_3_ exposure induced neurotoxicity in the rat CNS affecting mainly the hippocampus and cerebellum [284]. Another study [119] has shown that exposing mice to NaVO_3_; via i.p. induces the progressive accumulation of V, primarily in the OB, brainstem, and cerebellum, together with evidence that V crosses the BBB, morphologically alters the prefrontal cortex, and induces the degeneration of hippocampal CA_1_ pyramidal and cerebellar Purkinje cells, including astrogliosis and microgliosis. In addition, changes in catecholaminergic levels have been reported in different mouse brain structures after the ingestion of vanadate in drinking water [285].

Neurotoxicological studies have also investigated the behavioral effects of V exposure. Our rodent model research demonstrates that month-long intranasal V exposure induces olfactory and locomotor deficits [118]. Another study shows that eight consecutive weeks of oral exposure to V in rats induces motor and learning deficits [286]. Chronic i.p. administration of V in mice leads to memory deficits after 3 months of exposure and the effect persists until at least age 12 months [287]. V exposure through lactation reportedly induces neurotoxicity in the rat’s developing CNS that manifests as reduced muscular strength and locomotion in pups of both sexes [288]. Another study investigating V exposure via lactation for 15 and 22 days in neonatal mouse pups reported reduced locomotor activity and negative geotaxis [289]. In PINK-1 flies, chronic V exposure exacerbates motor deficits and reduces survival [290]. V exposure in humans may also cause CNS depression, tremors, neurasthenia, and other severe motor deficits, including vegetative symptoms. Other studies provide evidence that occupational V exposure alters neurobehavioral performance, including emotion, attention, cognition, short-term memory, reaction speed, accuracy, and coordination [291,292]. A case study reported that an individual exposed to V poisoning presented focal neurobiological deficits [293]. These findings suggest that occupational and environmental exposure to metals may play an important role in the etiopathogenesis of PD.

Additional studies show that chronic V exposure can also cause adverse respiratory system effects [294,295,296], hematotoxicity [297,298], thrombocytosis [299], renal toxicity [300], reproductive [301,302] and developmental toxicity [301], immunotoxicity, and mutagenicity. Cases involving mortality due to exposure to V compounds have also been reported [303].

Oxidative stress plays a prominent role in V toxicity, which has been associated with neurodegenerative diseases such as PD. V compounds induce ROS generation in the brain, which may contribute to the degeneration of DAergic neuronal cells of the SN, a hallmark of PD [120,121]. The ability of V to generate ROS in a Fenton-like reaction has been reported [304] and V can also generate ROS indirectly by releasing iron from intracellular stores [305]. Intracellular V compounds fluctuate between the anionic vanadate (V (+5); H_2_VO_4_^−^) and the cationic vanadyl (V (+4); hydrated VO^2^^+^) constantly occurring in the presence of ROS [305]. Intracellular antioxidants reduce vanadate to vanadyl, producing ROS in the process [306,307]. H_2_O_2_ oxidizes vanadyl in a Fenton-like reaction producing vanadate and hydroxyl radicals [308,309]. The oxidative stress resulting from high levels of vanadate can damage lipids, proteins, and nucleic acids. By binding to a protein’s cysteine residues, vanadate can disrupt the catalytic site of many enzymes such as protein tyrophosphatase (PTP) [310]. This inactivation mechanism can act as an irreversible inhibitor in the presence of H_2_O_2_, which transforms the cysteine-bound vanadate into pervanadate [265,310,311,312]. The prolonged inhibition of PTP activates protein tyrosine kinases (PTKs) that then activate the mitogen-activated protein kinase (MAPK) cascade, thereby initiating signal transduction [313,314,315] producing inflammatory cytokines [316].

V compounds induce mitochondrial oxidative stress that opens mitochondrial permeability transition pores, which leads to the collapse of mitochondrial membrane potential followed by the release of cytochrome c that culminates in mitochondrial-mediated cell apoptosis [122,123]. We too have demonstrated that the V-induced generation of ROS causes mitochondria to release cytochrome c, which signals the activation of caspases-9 and -3 [121]. Once activated, caspase-3 proteolytically activates PKCδ. Furthermore, we have found that inhibiting ROS, caspase activity, and PKCδ can attenuate V-induced DNA damage and apoptosis in DAergic neurons. This finding implies that V toxicity plays an important role in PKCδ-mediated DAergic neurotoxicity.

The dose-dependent effects of V on cellular processes may also depend on the presence of other metals. Thus, when V co-occurs as a mixture with one or more other metals like Mn, iron, selenium, magnesium, or lead, then characterizing their possible additive, synergistic, or antagonistic interactions would help to further elucidate the mechanisms underlying V’s neurotoxic effects.

## 9. Conclusions and Future Directions

PD is the most common movement disorder, impacting approximately 1% of people 65 years old or older. This review delineates the characteristics, neurotoxicity, neuropathology, and mechanisms of several key neurotoxic pesticides and metals, including MPTP, rotenone, PQ, DDT, dieldrin, Mn, and vanadium, that make DAergic neurons susceptible to PD. We also summarize current discoveries from epidemiological studies to decipher the correlation between environmental exposure in humans and neurological impairment. The pesticides reviewed here tend to share certain actions, i.e., inhibition of the mitochondrial respiratory chain and production of oxidative stress [317,318]. Antioxidants can be applied to attenuate their toxicity [319,320]. In addition to mitochondrial dysfunction, recent studies link microRNAs and pesticide neurotoxicity, revealing that microRNA dysregulation could be the novel mechanism underlying neurotoxic pesticide-induced neurotoxicity based on two conditions: (1) microRNAs sharing similar dysregulation functions with other types of epigenetic modification, and (2) the differential expression of microRNA occurring in PD patients [321,322,323,324]. Furthermore, exosomes are importantly involved in trafficking and cell-to-cell communication. This may have broad implications in the environmental stress response as exosomes can cross the BBB and communicate across various organs. The significance of toxicants entering the brain via the olfactory nerve, which bypasses the BBB, remains an exciting topic to explore for intervention strategies. The cellular responses to chemical exposure following the inhalation of environmental pollutants will depend on their different oxidation states and solubility, yet such parameters have not been adequately accounted for in existing human dose-response studies. Therefore, we need better epidemiology studies incorporating good tracing and management combined with complete occupational exposure histories with both behavioral and biochemical endpoints of neurotoxicity tailored to specific subgroups of PD patients. Considering the high societal cost of PD, advancing the environmental exposure assessment science and its integration with other approaches, including the epigenomic disease model toolbox, would help fill an unmet need.

## Figures and Tables

**Table 1:** Summary of the role of environmental risk factors in Parkinson’s disease.

Neurotoxins	Pathogenesis	Mechanism of Toxicity	Molecular and Cellular Alterations	References
MPTP	- DAergic neurons in SNPc ↓ - Striatal DA ↓ - Neurodegeneration in locus coeruleus - α-synuclein ↑ - Astrogliosis and microglial activation	- Crosses BBB and metabolized into the toxic cation MPP^+^ - Taken up by DA neurons via DAT - Concentrates in mitochondria and causes complex I defect - Reactive oxygen species ↑	- Mitochondrial fragmentation and mitophagy ↑ - Mitochondrial biogenesis ↓ - Intracellular Ca^2^^+^ ions ↑ - NFκB- dependent transactivation of iNOS, ↑ JNK and Bax, cytochrome c release, and caspase-3 and -9 activation leading to apoptosis	[13,35,36,37,38,39,40,41,42,43,44,45,46,47,48,49]
Rotenone	- DAergic neurodegeneration - α-synuclein rich LB-like inclusions - TH ↓ - Microglial activation and augmented neuroinflammation	- Crosses BBB - Mitochondrial complex I inhibition - Reactive oxygen species ↑ - Microtubule destabilizing activity	- ATP synthesis ↓ - Mitochondrial fragmentation ↑ and altered mitochondrial fission/fusion and biogenesis - Intracellular Ca^2^^+^ ions ↑ - Altered PI3K/Akt/GSK-3β/CREB signaling pathway - Mitochondrial impairment by Parkin ↓ and PINK1 ↑ - Caspase-3 and -9 activation leading to apoptosis	[50,51,52,53,54,55,56,57,58,59,60,61,62,63,64,65,66,67,68,69,70,71,72]
Paraquat	- DAergic neurons in SN ↓ - α-synuclein accumulation and aggregation	- Enters DAergic neurons via DAT - Generates oxygen-free radicals - Produces H202 via mitochondrial complex III	- Oxidative stress, cytochrome c release, caspase-3 and -9 activation, mitophagy and apoptosis - Ca^2+^ dyshomeostasis - Altered DA signaling pathway - Dysregulation of histone acetylation	[27,73,74,75,76,77,78,79]
DDT	Inconclusive evidence	- CNS excitation by sustained depolarization of nerve membrane - Mitochondrial complex II and V inhibition	- Cytosolic Ca^2+^ release and activation of apoptotic factors - Bcl2 ↓ and apoptosis induction through caspase-3 and -9 and GSK-3β - p53, NFκB and caspase-3 ↑ - Inhibition of DAT and VMAT	[18,80,81,82,83,84]
Dieldrin	- DAergic neurodegeneration	- Crosses BBB - May impair mitochondrial electron transport chain - Exacerbates MPTP and α-synuclein pre-formed fibril-mediated toxicity	- Mitochondrial dysfunction and oxidative stress - Caspase-3 activity ↑ and Fyn-mediated PKCδ activation followed by apoptosis - Ubiquitin-proteasome system dysfunction - Histone 3 and 4 acetylation ↑	[19,20,85,86,87,88,89,90]
Manganese	- DAergic neurons in SNPc ↓ - Striatal DA ↓ - Olfactory dysfunction - Microglial activation - Impaired neurogenesis	- Reactive oxygen species ↑	- Mitochondrial dysfunction and Ca^2+^ homeostasis disruption - Altered mitochondrial fission/fusion and biogenesis - Apoptosis-associated activation of PKCδ - Release of proinflammatory cytokines IL-1β, IL-6 and TNFα - Epigenetic dysregulation	[34,91,92,93,94,95,96,97,98,99,100,101,102,103,104,105,106,107,108,109,110,111,112,113,114]
Vanadium	- DAergic neurotransmission in olfactory bulb ↓ - Hippocampal CA_1_ pyramidal and cerebellar Purkinje cells ↓ - Changes in catecholaminergic levels - Astrogliosis and microgliosis	- Crosses BBB - Reactive oxygen species ↑	- Mitochondrial oxidative stress - Cytochrome c release, caspase-3 and -9 activation, and PKCδ activation leading to apoptosis	[115,116,117,118,119,120,121,122,123]

## Data Availability

Not applicable.

## References

[1] Sveinbjornsdottir S. (2016). The clinical symptoms of Parkinson’s disease. J. Neurochem..

[2] Schapira A.H.V., Chaudhuri K.R., Jenner P. (2017). Non-Motor features of Parkinson disease. Nat. Rev. Neurosci..

[3] Marras C., Beck J.C., Bower J.H., Roberts E., Ritz B., Ross G.W., Abbott R.D., Savica R., Van Den Eeden S.K., Willis A.W. (2018). Prevalence of Parkinson’s disease across North America. NPJ Parkinson’s Dis..

[4] Blauwendraat C., Nalls M.A., Singleton A.B. (2020). The genetic architecture of Parkinson’s disease. Lancet Neurol..

[5] Lunati A., Lesage S., Brice A. (2018). The genetic landscape of Parkinson’s disease. Rev. Neurol..

[6] Bellou V., Belbasis L., Tzoulaki I., Evangelou E., Ioannidis J.P. (2016). Environmental risk factors and Parkinson’s disease: An umbrella review of meta-analyses. Parkinsonism Relat Disord.

[7] Dick F.D., De Palma G., Ahmadi A., Scott N.W., Prescott G.J., Bennett J., Semple S., Dick S., Counsell C., Mozzoni P. (2007). Environmental risk factors for Parkinson’s disease and parkinsonism: The Geoparkinson study. Occup. Environ. Med..

[8] Pezzoli G., Cereda E. (2013). Exposure to pesticides or solvents and risk of Parkinson disease. Neurology.

[9] Priyadarshi A., Khuder S.A., Schaub E.A., Priyadarshi S.S. (2001). Environmental risk factors and Parkinson’s disease: A metaanalysis. Environ. Res..

[10] Breckenridge C.B., Berry C., Chang E.T., Sielken R.L., Mandel J.S. (2016). Association between Parkinson’s Disease and Cigarette Smoking, Rural Living, Well-Water Consumption, Farming and Pesticide Use: Systematic Review and Meta-Analysis. PLoS ONE.

[11] Silver M.R., Racette B.A., Dube U., Faust I.M., Nielsen S.S. (2020). Well Water and Parkinson’s Disease in Medicare Beneficiaries: A Nationwide Case-Control Study. J. Park. Dis..

[12] Dorsey E.R., Elbaz A., Nichols E., Abbasi N., Abd-Allah F., Abdelalim A., Adsuar J.C., Ansha M.G., Brayne C., Choi J.Y. (2018). Global, regional, and national burden of Parkinson’s disease, 1990–2016: A systematic analysis for the Global Burden of Disease Study 2016. Lancet Neurol..

[13] Langston J.W. (2017). The MPTP Story. J. Parkinson’s Dis..

[14] Tanner C.M., Kamel F., Ross G.W., Hoppin J.A., Goldman S.M., Korell M., Marras C., Bhudhikanok G.S., Kasten M., Chade A.R. (2011). Rotenone, paraquat, and Parkinson’s disease. Environ. Health Perspect..

[15] Giguère N., Burke Nanni S., Trudeau L.E. (2018). On Cell Loss and Selective Vulnerability of Neuronal Populations in Parkinson’s Disease. Front. Neurol..

[16] Minakaki G., Krainc D., Burbulla L.F. (2020). The Convergence of Alpha-Synuclein, Mitochondrial, and Lysosomal Pathways in Vulnerability of Midbrain Dopaminergic Neurons in Parkinson’s Disease. Front. Cell Dev. Biol..

[17] Rossi M., Scarselli M., Fasciani I., Maggio R., Giorgi F. (2017). Dichlorodiphenyltrichloroethane (DDT) induced extracellular vesicle formation: A potential role in organochlorine increased risk of Parkinson’s disease. Acta Neurobiol. Exp..

[18] Hatcher J.M., Delea K.C., Richardson J.R., Pennell K.D., Miller G.W. (2008). Disruption of dopamine transport by DDT and its metabolites. NeuroToxicology.

[19] Song C., Kanthasamy A., Anantharam V., Sun F., Kanthasamy A.G. (2010). Environmental Neurotoxic Pesticide Increases Histone Acetylation to Promote Apoptosis in Dopaminergic Neuronal Cells: Relevance to Epigenetic Mechanisms of Neurodegeneration. Mol. Pharmacol..

[20] Saminathan H., Asaithambi A., Anantharam V., Kanthasamy A.G., Kanthasamy A. (2011). Environmental neurotoxic pesticide dieldrin activates a non receptor tyrosine kinase to promote pkcδ-mediated dopaminergic apoptosis in a dopaminergic neuronal cell model. NeuroToxicology.

[21] Yan D., Zhang Y., Liu L., Shi N., Yan H. (2018). Pesticide exposure and risk of Parkinson’s disease: Dose-response meta-analysis of observational studies. Regul. Toxicol. Pharmacol..

[22] Hsieh T.-H., Chen J.-J.J., Chen L.-H., Chiang P.-T., Lee H.-Y. (2011). Time-Course gait analysis of hemiparkinsonian rats following 6-hydroxydopamine lesion. Behav. Brain Res..

[23] Sarkar S., Rokad D., Malovic E., Luo J., Harischandra D.S., Jin H., Anantharam V., Huang X., Lewis M., Kanthasamy A. (2019). Manganese activates NLRP3 inflammasome signaling and propagates exosomal release of ASC in microglial cells. Sci. Signal..

[24] Ngwa H.A., Ay M., Jin H., Anantharam V., Kanthasamy A., Kanthasamy A.G. (2017). Neurotoxicity of Vanadium. Adv. Neurobiol..

[25] Packer M., Miesel R., Murphy M. (1996). Exposure to the parkinsonian neurotoxin 1-methyl-4-phenylpyridinium (MPP+) and nitric oxide simultaneously causes cyclosporin A-sensitive mitochondrial calcium efflux and depolarisation. Biochem. Pharmacol..

[26] Sun Y., Sukumaran P., Selvaraj S., Cilz N.I., Schaar A., Lei S., Singh B.B. (2016). TRPM2 Promotes Neurotoxin MPP+/MPTP-Induced Cell Death. Mol. Neurobiol..

[27] Zaidi A., Fernandes D., Bean J.L., Michaelis M.L. (2009). Effects of paraquat-induced oxidative stress on the neuronal plasma membrane Ca^2+^-ATPase. Free Radic. Biol. Med..

[28] Singh Y., El-Hadidi M., Admard J., Wassouf Z., Schulze-Hentrich J., Kohlhofer U., Quintanilla-Martinez L., Huson D., Riess O., Casadei N. (2019). Enriched Environmental Conditions Modify the Gut Microbiome Composition and Fecal Markers of Inflammation in Parkinson’s Disease. Front. Neurosci..

[29] Ghaisas S., Maher J., Kanthasamy A. (2015). Gut microbiome in health and disease: Linking the microbiome–gut–brain axis and environmental factors in the pathogenesis of systemic and neurodegenerative diseases. Pharmacol. Ther..

[30] Delic V., Beck K.D., Pang K.C.H., Citron B.A. (2020). Biological links between traumatic brain injury and Parkinson’s disease. Acta Neuropathol. Commun..

[31] Sarkar S., Malovic E., Harishchandra D.S., Ghaisas S., Panicker N., Charli A., Palanisamy B.N., Rokad D., Jin H., Anantharam V. (2017). Mitochondrial impairment in microglia amplifies NLRP3 inflammasome proinflammatory signaling in cell culture and animal models of Parkinson’s disease. Npj Park. Dis..

[32] Gordon R., Neal M.L., Luo J., Langley M., Harischandra D.S., Panicker N., Charli A., Jin H., Anantharam V., Woodruff T.M. (2016). Prokineticin-2 upregulation during neuronal injury mediates a compensatory protective response against dopaminergic neuronal degeneration. Nat. Commun..

[33] Harischandra D.S., Rokad D., Neal M.L., Ghaisas S., Manne S., Sarkar S., Panicker N., Zenitsky G., Jin H., Lewis M. (2019). Manganese promotes the aggregation and prion-like cell-to-cell exosomal transmission of α-synuclein. Sci. Signal..

[34] Langley M., Ghaisas S., Ay M., Luo J., Palanisamy B.N., Jin H., Anantharam V., Kanthasamy A., Kanthasamy A.G. (2017). Manganese exposure exacerbates progressive motor deficits and neurodegeneration in the MitoPark mouse model of Parkinson’s disease: Relevance to gene and environment interactions in metal neurotoxicity. NeuroToxicology.

[35] Braak H., Ghebremedhin E., Rüb U., Bratzke H., Del Tredici K. (2004). Stages in the development of Parkinson’s disease-related pathology. Cell Tissue Res..

[36] Schildknecht S., Di Monte D.A., Pape R., Tieu K., Leist M. (2017). Tipping Points and Endogenous Determinants of Nigrostriatal Degeneration by MPTP. Trends Pharmacol. Sci..

[37] Kotake Y., Ohta S. (2003). MPP+ analogs acting on mitochondria and inducing neuro-degeneration. Curr. Med. Chem..

[38] Schober A. (2004). Classic toxin-induced animal models of Parkinson’s disease: 6-OHDA and MPTP. Cell Tissue Res..

[39] Przedborski S., Jackson-Lewis V., Naini A.B., Jakowec M., Petzinger G., Miller R., Akram M. (2001). The parkinsonian toxin 1-methyl-4-phenyl-1,2,3,6-tetrahydropyridine (MPTP): A technical review of its utility and safety. J. Neurochem..

[40] McGeer P.L., McGeer E.G. (2008). Glial reactions in Parkinson’s disease. Mov. Disord..

[41] Han N.-R., Kim Y.-K., Ahn S., Hwang T.-Y., Lee H., Park H.-J. (2021). A Comprehensive Phenotype of Non-motor Impairments and Distribution of Alpha-Synuclein Deposition in Parkinsonism-Induced Mice by a Combination Injection of MPTP and Probenecid. Front. Aging Neurosci..

[42] Carbone D.L., Popichak K.A., Moreno J.A., Safe S., Tjalkens R.B. (2008). Suppression of 1-Methyl-4-phenyl-1,2,3,6-tetrahydropyridine-Induced Nitric-Oxide Synthase 2 Expression in Astrocytes by a Novel Diindolylmethane Analog Protects Striatal Neurons against Apoptosis. Mol. Pharmacol..

[43] Saporito M., Brown E.M., Miller M.S., Carswell S. (1999). CEP-1347/KT-7515, an inhibitor of c-jun N-terminal kinase activation, attenuates the 1-methyl-4-phenyl tetrahydropyridine-mediated loss of nigrostriatal dopaminergic neurons In vivo. J. Pharmacol. Exp. Ther..

[44] Vila M., Jackson-Lewis V., Vukosavic S., Djaldetti R., Liberatore G., Offen D., Korsmeyer S.J., Przedborski S. (2001). Bax ablation prevents dopaminergic neurodegeneration in the 1-methyl- 4-phenyl-1,2,3,6-tetrahydropyridine mouse model of Parkinson’s disease. Proc. Natl. Acad. Sci. USA.

[45] Viswanath V., Wu Y., Boonplueang R., Chen S., Stevenson F.F., Yantiri F., Yang L., Beal M.F., Andersen J.K. (2001). Caspase-9 Activation Results in Downstream Caspase-8 Activation and Bid Cleavage in 1-Methyl-4-Phenyl-1,2,3,6-Tetrahydropyridine-Induced Parkinson’s Disease. J. Neurosci..

[46] Zhang Q.S., Heng Y., Mou Z., Huang J.Y., Yuan Y.H., Chen N.H. (2017). Reassessment of subacute MPTP-treated mice as animal model of Parkinson’s disease. Acta Pharmacol. Sin..

[47] Chuang J.-I., Pan I.-L., Hsieh C.-Y., Huang C.-Y., Chen P.-C., Shin J.W. (2016). Melatonin prevents the dynamin-related protein 1-dependent mitochondrial fission and oxidative insult in the cortical neurons after 1-methyl-4-phenylpyridinium treatment. J. Pineal Res..

[48] Wang X., Su B., Liu W., He X., Gao Y., Castellani R.J., Perry G., Smith M.A., Zhu X. (2011). DLP1-dependent mitochondrial fragmentation mediates 1-methyl-4-phenylpyridinium toxicity in neurons: Implications for Parkinson’s disease. Aging Cell.

[49] Rao S.P., Sharma N., Kalivendi S.V. (2020). Embelin averts MPTP-induced dysfunction in mitochondrial bioenergetics and biogenesis via activation of SIRT1. Biochim. Biophys. Acta.

[50] Palmer G., Horgan D.J., Tisdale H.O., Singer T.P., Beinert H. (1968). Studies on the respiratory chain-linked reduced nicotinamide adenine dinucleotide dehydrogenase. XIV. Location of the sites of inhibition of rotenone, barbiturates, and piericidin by means of electron paramagnetic resonance spectroscopy. J. Biol. Chem..

[51] Li N., Ragheb K., Lawler G., Sturgis J., Rajwa B., Melendez J.A., Robinson J.P. (2003). Mitochondrial complex I inhibitor rotenone induces apoptosis through enhancing mitochondrial reactive oxygen species production. J. Biol. Chem..

[52] Huang C.-W., Lin K.-M., Hung T.-Y., Chuang Y.-C., Wu S.-N. (2018). Multiple Actions of Rotenone, an Inhibitor of Mitochondrial Respiratory Chain, on Ionic Currents and Miniature End-Plate Potential in Mouse Hippocampal (mHippoE-14) Neurons. Cell. Physiol. Biochem..

[53] Passmore J.B., Pinho S., Gomez-Lazaro M., Schrader M. (2017). The respiratory chain inhibitor rotenone affects peroxisomal dynamics via its microtubule-destabilising activity. Histochem. Cell Biol..

[54] Johnson M.E., Bobrovskaya L. (2015). An update on the rotenone models of Parkinson’s disease: Their ability to reproduce the features of clinical disease and model gene–environment interactions. NeuroToxicology.

[55] Betarbet R., Sherer T.B., MacKenzie G., Garcia-Osuna M., Panov A.V., Greenamyre J.T. (2000). Chronic systemic pesticide exposure reproduces features of Parkinson’s disease. Nat. Neurosci..

[56] Wrangel C.V., Schwabe K., John N., Krauss J.K., Alam M. (2015). The rotenone-induced rat model of Parkinson’s disease: Behavioral and electrophysiological findings. Behav. Brain Res..

[57] Zhang Z.N., Zhang J.S., Xiang J., Yu Z.H., Zhang W., Cai M., Li X.T., Wu T., Li W.W., Cai D.F. (2017). Subcutaneous rotenone rat model of Parkinson’s disease: Dose exploration study. Brain Res..

[58] Cannon J.R., Tapias V., Na H.M., Honick A.S., Drolet R.E., Greenamyre J.T. (2009). A highly reproducible rotenone model of Parkinson’s disease. Neurobiol. Dis..

[59] Carriere C.H., Kang N.H., Niles L.P. (2016). Chronic low-dose melatonin treatment maintains nigrostriatal integrity in an intrastriatal rotenone model of Parkinson’s disease. Brain Res..

[60] Chu C.T., Ji J., Dagda R.K., Jiang J.F., Tyurina Y.Y., Kapralov A.A., Tyurin V.A., Yanamala N., Shrivastava I.H., Mohammadyani D. (2013). Cardiolipin externalization to the outer mitochondrial membrane acts as an elimination signal for mitophagy in neuronal cells. Nat. Cell Biol..

[61] Chu C.T., Bayır H., Kagan V.E. (2014). LC3 binds externalized cardiolipin on injured mitochondria to signal mitophagy in neurons: Implications for Parkinson disease. Autophagy.

[62] Wu X., Liang Y., Jing X., Lin D., Chen Y., Zhou T., Peng S., Zheng D., Zeng Z., Lei M. (2018). Rifampicin Prevents SH-SY5Y Cells from Rotenone-Induced Apoptosis via the PI3K/Akt/GSK-3β/CREB Signaling Pathway. Neurochem. Res..

[63] Rokad D., Ghaisas S., Harischandra D., Jin H., Anantharam V., Kanthasamy A., Kanthasamy A.G. (2016). Role of neurotoxicants and traumatic brain injury in α-synuclein protein misfolding and aggregation. Brain Res. Bull..

[64] Ramalingam M., Huh Y.-J., Lee Y.-I. (2019). The Impairments of α-Synuclein and Mechanistic Target of Rapamycin in Rotenone-Induced SH-SY5Y Cells and Mice Model of Parkinson’s Disease. Front. Neurosci..

[65] Angeline M.S., Chaterjee P., Anand K., Ambasta R.K., Kumar P. (2012). Rotenone-Induced parkinsonism elicits behavioral impairments and differential expression of parkin, heat shock proteins and caspases in the rat. Neuroscience.

[66] Gao H.M., Liu B., Hong J.S. (2003). Critical role for microglial NADPH oxidase in rotenone-induced degeneration of dopaminergic neurons. J. Neurosci. Off. J. Soc. Neurosci..

[67] Drechsel D.A., Patel M. (2008). Role of reactive oxygen species in the neurotoxicity of environmental agents implicated in Parkinson’s disease. Free. Radic. Biol. Med..

[68] Kanthasamy A., Jin H., Charli A., Vellareddy A., Kanthasamy A. (2019). Environmental neurotoxicant-induced dopaminergic neurodegeneration: A potential link to impaired neuroinflammatory mechanisms. Pharmacol. Ther..

[69] Lawana V., Singh N., Sarkar S., Charli A., Jin H., Anantharam V., Kanthasamy A.G., Kanthasamy A. (2017). Involvement of c-Abl Kinase in Microglial Activation of NLRP3 Inflammasome and Impairment in Autolysosomal System. J. Neuroimmune Pharmacol. Off. J. Soc. NeuroImmune Pharmacol..

[70] Liu C., Ye Y., Zhou Q., Zhang R., Zhang H., Liu W., Xu C., Liu L., Huang S., Chen L. (2016). Crosstalk between Ca^2+^ signaling and mitochondrial H_2_O_2_ is required for rotenone inhibition of mTOR signaling pathway leading to neuronal apoptosis. Oncotarget.

[71] Peng K., Tao Y., Zhang J., Wang J., Ye F., Dan G., Zhao Y., Cai Y., Zhao J., Wu Q. (2016). Resveratrol Regulates Mitochondrial Biogenesis and Fission/Fusion to Attenuate Rotenone-Induced Neurotoxicity. Oxidative Med. Cell. Longev..

[72] Peng K., Yang L., Wang J., Ye F., Dan G., Zhao Y., Cai Y., Cui Z., Ao L., Liu J. (2016). The Interaction of Mitochondrial Biogenesis and Fission/Fusion Mediated by PGC-1α Regulates Rotenone-Induced Dopaminergic Neurotoxicity. Mol. Neurobiol..

[73] Castello P.R., Drechsel D.A., Patel M. (2007). Mitochondria Are a Major Source of Paraquat-induced Reactive Oxygen Species Production in the Brain. J. Biol. Chem..

[74] Rappold P.M., Cui M., Chesser A.S., Tibbett J., Grima J.C., Duan L., Sen N., Javitch J.A., Tieu K. (2011). Paraquat neurotoxicity is mediated by the dopamine transporter and organic cation transporter-3. Proc. Natl. Acad. Sci. USA.

[75] McCarthy S., Somayajulu M., Sikorska M., Borowy-Borowski H., Pandey S. (2004). Paraquat induces oxidative stress and neuronal cell death; neuroprotection by water-soluble Coenzyme Q10. Toxicol. Appl. Pharmacol..

[76] Somayajulu-Niţu M., Sandhu J.K., Cohen J., Sikorska M., Sridhar T., Matei A., Borowy-Borowski H., Pandey S. (2009). Paraquat induces oxidative stress, neuronal loss in substantia nigra region and Parkinsonism in adult rats: Neuroprotection and amelioration of symptoms by water-soluble formulation of Coenzyme Q10. BMC Neurosci..

[77] Song C., Kanthasamy A., Jin H., Anantharam V. (2011). Paraquat induces epigenetic changes by promoting histone acetylation in cell culture models of dopaminergic degeneration. NeuroToxicology.

[78] See W.Z.C., Naidu R., Tang K.S. (2022). Cellular and Molecular Events Leading to Paraquat-Induced Apoptosis: Mechanistic Insights into Parkinson’s Disease Pathophysiology. Mol. Neurobiol..

[79] Manning-Bog A.B., McCormack A.L., Li J., Uversky V.N., Fink A.L., Di Monte D.A. (2002). The herbicide paraquat causes up-regulation and aggregation of alpha-synuclein in mice: Paraquat and alpha-synuclein. J. Biol. Chem..

[80] Harada T., Takeda M., Kojima S., Tomiyama N. (2016). Toxicity and Carcinogenicity of Dichlorodiphenyltrichloroethane (DDT). Toxicol. Res..

[81] Zhao M., Wang C., Zhang C., Wen Y., Liu W. (2012). Enantioselective Cytotoxicity Profile of o,p’-DDT in PC 12 Cells. PLoS ONE.

[82] Kajta M., Litwa E., Rzemieniec J., Wnuk A., Lason W., Zelek-Molik A., Nalepa I., Grzegorzewska-Hiczwa M., Tokarski K., Golas A. (2014). Isomer-nonspecific action of dichlorodiphenyltrichloroethane on aryl hydrocarbon receptor and G-protein-coupled receptor 30 intracellular signaling in apoptotic neuronal cells. Mol. Cell. Endocrinol..

[83] Ferreira F.M., Madeira V.M., Moreno A.J. (1997). Interactions of 2,2-bis(p-chlorophenyl)-1,1-dichloroethylene with Mitochondrial Oxidative Phosphorylation. Biochem. Pharmacol..

[84] Moreno A.J., Madeira V.M. (1991). Mitochondrial bioenergetics as affected by DDT. Biochim. Biophys. Acta.

[85] Kanthasamy A.G., Kitazawa M., Kanthasamy A., Anantharam V. (2005). Dieldrin-Induced Neurotoxicity: Relevance to Parkinson’s Disease Pathogenesis. NeuroToxicology.

[86] American Chemical Society Pesticide Exposure Could Increase Risk of Early Onset of Parkinson’s Disease. https://www.sciencedaily.com/releases/2006/09/060914194700.htm.

[87] Bergen W.G. (1971). The In Vitro Effect of Dieldrin on Respiration of Rat Liver Mitochondria. Exp. Biol. Med..

[88] Hatcher J.M., Richardson J., Guillot T.S., McCormack A.L., Di Monte D., Jones D.P., Pennell K., Miller G.W. (2007). Dieldrin exposure induces oxidative damage in the mouse nigrostriatal dopamine system. Exp. Neurol..

[89] Kanthasamy A.G., Kitazawa M., Yang Y., Anantharam V., Kanthasamy A. (2008). Environmental neurotoxin dieldrin induces apoptosis via caspase-3-dependent proteolytic activation of protein kinase C delta (PKCdelta): Implications for neurodegeneration in Parkinson’s disease. Mol. Brain.

[90] Sun F., Anantharam V., Latchoumycandane C., Kanthasamy A., Kanthasamy A. (2005). Dieldrin Induces Ubiquitin-Proteasome Dysfunction in α-Synuclein Overexpressing Dopaminergic Neuronal Cells and Enhances Susceptibility to Apoptotic Cell Death. J. Pharmacol. Exp. Ther..

[91] Harischandra D.S., Ghaisas S., Zenitsky G., Jin H., Kanthasamy A., Anantharam V., Kanthasamy A.G. (2019). Manganese-Induced Neurotoxicity: New Insights Into the Triad of Protein Misfolding, Mitochondrial Impairment, and Neuroinflammation. Front. Neurosci..

[92] Fitsanakis V.A., Au C., Erikson K.M., Aschner M. (2006). The effects of manganese on glutamate, dopamine and gamma-aminobutyric acid regulation. Neurochem. Int..

[93] Kwakye G.F., Paoliello M.M., Mukhopadhyay S., Bowman A.B., Aschner M. (2015). Manganese-Induced Parkinsonism and Parkinson’s Disease: Shared and Distinguishable Features. Int. J. Environ. Res. Public Health.

[94] Gonzalez-Cuyar L.F., Nelson G., Criswell S.R., Ho P., Lonzanida J.A., Checkoway H., Seixas N., Gelman B.B., Evanoff B.A., Murray J. (2013). Quantitative neuropathology associated with chronic manganese exposure in South African mine workers. NeuroToxicology.

[95] Antunes M.B., Bowler R., Doty R.L. (2007). San Francisco/Oakland Bay Bridge Welder Study: Olfactory function. Neurology.

[96] Bowler R.M., Roels H.A., Nakagawa S., Drezgic M., Diamond E., Park R., Koller W., Mergler D., Bouchard M., Smith D. (2007). Dose-effect relationships between manganese exposure and neurological, neuropsychological and pulmonary function in confined space bridge welders. Occup. Environ. Med..

[97] Bowler R.M., Gocheva V., Harris M., Ngo L., Abdelouahab N., Wilkinson J., Doty R.L., Park R., Roels H.A. (2011). Prospective study on neurotoxic effects in manganese-exposed bridge construction welders. NeuroToxicology.

[98] Stagg C.J., Bestmann S., Constantinescu A.O., Moreno L.M., Allman C., Mekle R., Woolrich M., Near J., Johansen-Berg H., Rothwell J. (2011). Relationship between physiological measures of excitability and levels of glutamate and GABA in the human motor cortex. J. Physiol..

[99] Guarneros M., Ortiz-Romo N., Alcaraz-Zubeldia M., Drucker-Colín R., Hudson R. (2013). Nonoccupational Environmental Exposure to Manganese is Linked to Deficits in Peripheral and Central Olfactory Function. Chem. Senses.

[100] Lucchini R.G., Guazzetti S., Zoni S., Donna F., Peter S., Zacco A., Salmistraro M., Bontempi E., Zimmerman N.J., Smith D.R. (2012). Tremor, olfactory and motor changes in Italian adolescents exposed to historical ferro-manganese emission. NeuroToxicology.

[101] Iannilli E., Gasparotti R., Hummel T., Zoni S., Benedetti C., Fedrighi C., Tang C.Y., Van Thriel C., Lucchini R.G. (2016). Effects of Manganese Exposure on Olfactory Functions in Teenagers: A Pilot Study. PLoS ONE.

[102] Rolle-McFarland D., Liu Y., Mostafaei F., Zauber S.E., Zhou Y., Li Y., Fan Q., Zheng W., Nie L.H., Wells E.M. (2019). The association of bone, fingernail and blood manganese with cognitive and olfactory function in Chinese workers. Sci. Total Environ..

[103] Aschner M., Erikson K.M., Herrero Hernández E., Hernández E.H., Tjalkens R. (2009). Manganese and its role in Parkinson’s disease: From transport to neuropathology. Neuromolecular Med..

[104] Ngwa H.A., Kanthasamy A., Gu Y., Fang N., Anantharam V., Kanthasamy A.G. (2011). Manganese nanoparticle activates mitochondrial dependent apoptotic signaling and autophagy in dopaminergic neuronal cells. Toxicol. Appl. Pharmacol..

[105] Tarale P., Daiwile A.P., Sivanesan S., Stöger R., Bafana A., Naoghare P.K., Parmar D., Chakrabarti T., Krishnamurthi K. (2018). Manganese exposure: Linking down-regulation of miRNA-7 and miRNA-433 with α-synuclein overexpression and risk of idiopathic Parkinson’s disease. Toxicol. In Vitro.

[106] Guo Z., Zhang Z., Wang Q., Zhang J., Wang L., Zhang Q., Li H., Wu S. (2018). Manganese chloride induces histone acetylation changes in neuronal cells: Its role in manganese-induced damage. NeuroToxicology.

[107] Tarale P., Stöger R., Bafana A., Parmar D., Chakrabarti T., Kannan K., Daiwile A.P., Naoghare P.K., Sivanesan S. (2016). Global DNA methylation profiling of manganese-exposed human neuroblastoma SH-SY5Y cells reveals epigenetic alterations in Parkinson’s disease-associated genes. Arch. Toxicol..

[108] Wang L., Shiraki A., Itahashi M., Akane H., Abe H., Mitsumori K., Shibutani M. (2013). Aberration in Epigenetic Gene Regulation in Hippocampal Neurogenesis by Developmental Exposure to Manganese Chloride in Mice. Toxicol. Sci..

[109] Hou L., Zhang X., Tarantini L., Nordio F., Bonzini M., Angelici L., Marinelli B., Rizzo G., Cantone L., Apostoli P. (2011). Ambient PM exposure and DNA methylation in tumor suppressor genes: A cross-sectional study. Part. Fibre Toxicol..

[110] Lim J., Bang Y., Choi H.J. (2018). Abnormal hippocampal neurogenesis in Parkinson’s disease: Relevance to a new therapeutic target for depression with Parkinson’s disease. Arch. Pharmacal Res..

[111] Han M.-H., Lee E.-H., Koh S.-H. (2016). Current Opinion on the Role of Neurogenesis in the Therapeutic Strategies for Alzheimer Disease, Parkinson Disease, and Ischemic Stroke; Considering Neuronal Voiding Function. Int. Neurourol. J..

[112] Adamson S.X.-F., Shen X., Jiang W., Lai V., Wang X., Shannahan J.H., Cannon J.R., Chen J., Zheng W. (2018). Subchronic Manganese Exposure Impairs Neurogenesis in the Adult Rat Hippocampus. Toxicol. Sci..

[113] Ijomone O.M., Aluko O.M., Okoh C.O.A., Martins A.C., Aschner M. (2019). Role for calcium signaling in manganese neurotoxicity. J. Trace Elem. Med. Biol. Organ Soc. Miner. Trace Elem. (GMS).

[114] Sarkar S., Malovic E., Harischandra D.S., Ngwa H.A., Ghosh A., Hogan C., Rokad D., Zenitsky G., Jin H., Anantharam V. (2017). Manganese exposure induces neuroinflammation by impairing mitochondrial dynamics in astrocytes. NeuroToxicology.

[115] Avila-Costa M.R., Flores E.M., Colin-Barenque L., Ordoñez J.L., Gutiérrez A.L., Niño-Cabrera H.G., Mussali-Galante P., Fortoul T.I. (2004). Nigrostriatal Modifications After Vanadium Inhalation: An Immunocytochemical and Cytological Approach. Neurochem. Res..

[116] Avila-Costa M.R., Fortoul T., Niño-Cabrera G., Colín-Barenque L., Bizarro-Nevares P., Gutiérrez-Valdez A.L., Ordóñez-Librado J.L., Rodríguez-Lara V., Mussali-Galante P., Díaz-Bech P. (2006). Hippocampal cell alterations induced by the inhalation of vanadium pentoxide (V2O5) promote memory deterioration. NeuroToxicology.

[117] Avila-Costa M.R., Colín-Barenque L., Zepeda-Rodríguez A., Antuna S.B., Saldivar O.L., Espejel-Maya G., Mussali-Galante P., del Carmen Avila-Casado M., Reyes-Olivera A., Anaya-Martinez V. (2005). Ependymal epithelium disruption after vanadium pentoxide inhalation. A mice experimental model. Neurosci. Lett..

[118] Ngwa H.A., Kanthasamy A., Jin H., Anantharam V., Kanthasamy A.G. (2013). Vanadium exposure induces olfactory dysfunction in an animal model of metal neurotoxicity. NeuroToxicology.

[119] Folarin O.R., Snyder A.M., Peters D.G., Olopade F., Connor J.R., Olopade J.O. (2017). Brain Metal Distribution and Neuro-Inflammatory Profiles after Chronic Vanadium Administration and Withdrawal in Mice. Front. Neuroanat..

[120] Cuesta S., Francés D., García G.B. (2011). ROS formation and antioxidant status in brain areas of rats exposed to sodium metavanadate. Neurotoxicology Teratol..

[121] Afeseh Ngwa H., Kanthasamy A., Anantharam V., Song C., Witte T., Houk R., Kanthasamy A.G. (2009). Vanadium induces dopaminergic neurotoxicity via protein kinase Cdelta dependent oxidative signaling mechanisms: Relevance to etiopathogenesis of Parkinson’s disease. Toxicol. Appl. Pharmacol..

[122] Zhao Y., Ye L., Liu H., Xia Q., Zhang Y., Yang X., Wang K. (2010). Vanadium compounds induced mitochondria permeability transition pore (PTP) opening related to oxidative stress. J. Inorg. Biochem..

[123] Hosseini M.-J., Shaki F., Ghazi-Khansari M., Pourahmad J. (2013). Toxicity of vanadium on isolated rat liver mitochondria: A new mechanistic approach. Metallomics.

[124] Tysnes O.B., Storstein A. (2017). Epidemiology of Parkinson’s disease. J. Neural Transm..

[125] Langston J.W., Ballard P., Tetrud J.W., Irwin I. (1983). Chronic Parkinsonism in humans due to a product of meperidine-analog synthesis. Science.

[126] Dauer W., Przedborski S. (2003). Parkinson’s disease: Mechanisms and models. Neuron.

[127] Sugumar M., Sevanan M., Sekar S. (2018). Neuroprotective effect of naringenin against MPTP-induced oxidative stress. Int. J. Neurosci..

[128] Del Zompo M., Piccardi M., Ruiu S., Corsini G., Vaccari A. (1991). High-affinity binding of [3H]1-methyl-4-phenyl-2,3-dihydropyridinium ion to mouse striatal membranes: Putative vesicular location. Eur. J. Pharmacol..

[129] Del Zompo M., Piccardi M., Ruiu S., Corsini G., Vaccari A. (1992). Characterization of a putatively vesicular binding site for [3H]MPP+ in mouse striatal membranes. Brain Res..

[130] Peter D., Jimenez J., Liu Y., Kim J., Edwards R.H. (1994). The chromaffin granule and synaptic vesicle amine transporters differ in substrate recognition and sensitivity to inhibitors. J. Biol. Chem..

[131] Lotharius J., Brundin P. (2002). Pathogenesis of Parkinson’s disease: Dopamine, vesicles and alpha-synuclein. Nat. Rev. Neurosci..

[132] Munoz-Manchado A.B., Villadiego J., Romo-Madero S., Suarez-Luna N., Bermejo-Navas A., Rodriguez-Gomez J.A., Garrido-Gil P., Labandeira-Garcia J.L., Echevarria M., Lopez-Barneo J. (2016). Chronic and progressive Parkinson’s disease MPTP model in adult and aged mice. J. Neurochem..

[133] Masilamoni G.J., Smith Y. (2017). Chronic MPTP administration regimen in monkeys: A model of dopaminergic and non-dopaminergic cell loss in Parkinson’s disease. J. Neural Transm..

[134] (1933). Rotenone as an Insecticide. Nature.

[135] Isman M. (2006). Botanical insecticides, deterrents, and repellents in modern agriculture and an increasingly regulated world. Annu. Rev. Entomol..

[136] Soloway S.B. (1976). Naturally occurring insecticides. Environ. Health Perspect..

[137] World Health Organization (1992). International Programme on Chemical Safety, Rotenone: Health and Safety Guide.

[138] Nandipati S., Litvan I. (2016). Environmental Exposures and Parkinson’s Disease. Int. J. Environ. Res. Public Health.

[139] US Environmental Protection Agency (2007). Reregistration Eligibility Decision for Rotenone.

[140] Newsome W.H., Shields J.B. (1980). Residues of rotenone and rotenolone on lettuce and tomato fruit after treatment in the field with rotenone formulations. J. Agric. Food Chem..

[141] Zhou Y., Zhang N., Wang K., Li W., Li H., Zhang Z. (2013). Dissipation and Residue of Rotenone in Cabbage and Soil Under Field Conditions. Bull. Environ. Contam. Toxicol..

[142] Cabras P., Caboni P., Cabras M., Angioni A., Russo M. (2002). Rotenone residues on olives and in olive oil. J. Agric. Food Chem..

[143] Cavoski I., Caboni P., Sarais G., Miano T. (2008). Degradation and persistence of rotenone in soils and influence of temperature variations. J. Agric. Food Chem..

[144] US Environmental Protection Agency (2011). Product Cancellation Order for Certain Pesticide Registrations. Fed. Regist..

[145] US Environmental Protection Agency (2012). Tolerance Actions. Fed. Regist..

[146] Baker B. Rotenone Use in Organic Farming|Hygeia Analytics. https://hygeia-analytics.com/2017/01/04/rotenone-use-in-organic-farming/.

[147] Imamura K., Takeshima T., Kashiwaya Y., Nakaso K., Nakashima K. (2006). D-beta-hydroxybutyrate protects dopaminergic SH-SY5Y cells in a rotenone model of Parkinson’s disease. J. Neurosci. Res..

[148] Srivastava P., Panda D. (2007). Rotenone inhibits mammalian cell proliferation by inhibiting microtubule assembly through tubulin binding. FEBS J..

[149] Schapira A.H. (2008). Mitochondria in the aetiology and pathogenesis of Parkinson’s disease. Lancet Neurol..

[150] Dhillon A.S., Tarbutton G.L., Levin J.L., Plotkin G.M., Lowry L.K., Nalbone J.T., Shepherd S. (2008). Pesticide/Environmental Exposures and Parkinson’s Disease in East Texas. J. Agromed..

[151] Spivey A. (2011). Rotenone and Paraquat Linked to Parkinson’s Disease: Human Exposure Study Supports Years of Animal Studies. Environ. Health Perspect..

[152] Furlong M., Tanner C.M., Goldman S.M., Bhudhikanok G.S., Blair A., Chade A., Comyns K., Hoppin J.A., Kasten M., Korell M. (2015). Protective glove use and hygiene habits modify the associations of specific pesticides with Parkinson’s disease. Environ. Int..

[153] Pouchieu C., Piel C., Carles C., Gruber A., Helmer C., Tual S., Marcotullio E., Lebailly P., Baldi I. (2018). Pesticide use in agriculture and Parkinson’s disease in the AGRICAN cohort study. Int. J. Epidemiol..

[154] Zeng X.-S., Geng W.-S., Jia J.-J. (2018). Neurotoxin-Induced Animal Models of Parkinson Disease: Pathogenic Mechanism and Assessment. ASN Neuro.

[155] Inden M., Kitamura Y., Abe M., Tamaki A., Takata K., Taniguchi T. (2011). Parkinsonian rotenone mouse model: Reevaluation of long-term administration of rotenone in C57BL/6 mice. Biol. Pharm. Bull..

[156] Ren Y., Liu W., Jiang H., Jiang Q., Feng J. (2005). Selective vulnerability of dopaminergic neurons to microtubule depolymerization. J. Biol. Chem..

[157] Xiong N., Xiong J., Jia M., Liu L., Zhang X., Chen Z., Huang J., Zhang Z., Hou L., Luo Z. (2013). The role of autophagy in Parkinson’s disease: Rotenone-based modeling. Behav. Brain Funct. BBF.

[158] Tabata Y., Imaizumi Y., Sugawara M., Andoh-Noda T., Banno S., Chai M., Sone T., Yamazaki K., Ito M., Tsukahara K. (2018). T-type Calcium Channels Determine the Vulnerability of Dopaminergic Neurons to Mitochondrial Stress in Familial Parkinson Disease. Stem Cell Rep..

[159] Yuan Y.H., Yan W.F., Sun J.D., Huang J.Y., Mu Z., Chen N.H. (2015). The molecular mechanism of rotenone-induced α-synuclein aggregation: Emphasizing the role of the calcium/GSK3β pathway. Toxicol. Lett..

[160] Silva B.A., Einarsdóttir O., Fink A.L., Uversky V.N. (2013). Biophysical Characterization of α-Synuclein and Rotenone Interaction. Biomolecules.

[161] Arnold B., Cassady S., Van Laar V.S., Berman S.B. (2011). Integrating multiple aspects of mitochondrial dynamics in neurons: Age-related differences and dynamic changes in a chronic rotenone model. Neurobiol. Dis..

[162] Zhang X., Du L., Zhang W., Yang Y., Zhou Q., Du G. (2017). Therapeutic effects of baicalein on rotenone-induced Parkinson’s disease through protecting mitochondrial function and biogenesis. Sci. Rep..

[163] Barsoum M.J., Yuan H., Gerencser A.A., Liot G., Kushnareva Y., Gräber S., Kovacs I., Lee W.D., Waggoner J., Cui J. (2006). Nitric oxide-induced mitochondrial fission is regulated by dynamin-related GTPases in neurons. EMBO J..

[164] Staiff D.C., Comer S.W., Armstrong J.F., Wolfe H.R. (1975). Exposure to the herbicide, paraquat. Bull Environ. Contam. Toxicol..

[165] Smith J. (1988). Paraquat Poisoning by Skin Absorption: A Review. Hum. Toxicol..

[166] Baharudin M.R., Sahid I.B., Noor M.A.B.M., Sulaiman N., Othman F. (2011). Pesticide risk assessment: A study on inhalation and dermal exposure to 2,4-D and paraquat among Malaysian paddy farmers. J. Environ. Sci. Health Part B.

[167] Dinis-Oliveira R.J., Remião F., Carmo H., Duarte J.A., Navarro A.S., Bastos M.L., Carvalho F. (2006). Paraquat exposure as an etiological factor of Parkinson’s disease. Neurotoxicology.

[168] Wang X.H., Souders C.L., Zhao Y.H., Martyniuk C.J. (2018). Paraquat affects mitochondrial bioenergetics, dopamine system expression, and locomotor activity in zebrafish (Danio rerio). Chemosphere.

[169] Huang M., Li Y., Wu K., Yan W., Tian T., Wang Y., Yang H. (2019). Paraquat modulates microglia M1/M2 polarization via activation of TLR4-mediated NF-κB signaling pathway. Chem. Biol. Interact..

[170] McCormack A., Atienza J., Langston J., Di Monte D. (2006). Decreased susceptibility to oxidative stress underlies the resistance of specific dopaminergic cell populations to paraquat-induced degeneration. Neuroscience.

[171] Konthonbut P., Kongtip P., Nankongnab N., Tipayamongkholgul M., Yoosook W., Woskie S. (2018). Paraquat Exposure of Pregnant Women and Neonates in Agricultural Areas in Thailand. Int. J. Environ. Res. Public Health.

[172] Goldman S.M., Kamel F., Ross G.W., Bhudhikanok G.S., Hoppin J.A., Korell M., Marras C., Meng C., Umbach D.M., Kasten M. (2012). Genetic modification of the association of paraquat and Parkinson’s disease. Mov. Disord..

[173] Thiruchelvam M., McCormack A., Richfield E.K., Baggs R.B., Tank A.W., Di Monte D.A., Cory-Slechta D.A. (2003). Age-Related irreversible progressive nigrostriatal dopaminergic neurotoxicity in the paraquat and maneb model of the Parkinson’s disease phenotype. Eur. J. Neurosci..

[174] Laden F., Neas L.M., Spiegelman D., Hankinson S.E., Willett W.C., Ireland K., Wolff M.S., Hunter D.J. (1999). Predictors of plasma concentrations of DDE and PCBs in a group of U.S. women. Env. Health Perspect.

[175] Whitmore R.W., Immerman F.W., Camann D.E., Bond A.E., Lewis R.G., Schaum J.L. (1994). Non-occupational exposures to pesticides for residents of two U.S. cities. Arch. Environ. Contam. Toxicol..

[176] Hjelmborg P.S., Andreassen T.K., Bonefeld-Jørgensen E.C. (2008). Cellular uptake of lipoproteins and persistent organic compounds—An update and new data. Environ. Res..

[177] Rossi M., Scarselli M., Fasciani I., Marampon F., Maggio R., Pietrantoni I. (2018). Dichlorodiphenyltrichloroethane, an old pesticide with a new mechanism of toxicity. Curr. Top. Pharmacol..

[178] Costa L.G. (2015). The neurotoxicity of organochlorine and pyrethroid pesticides. Handb. Clin. Neurol..

[179] Richardson J., Roy A., Shalat S.L., Von Stein R.T., Hossain M.M., Buckley B., Gearing M., Levey A.I., German D.C. (2014). Elevated Serum Pesticide Levels and Risk for Alzheimer Disease. JAMA Neurol..

[180] World Health Organization (1990). Public Health Impact of Pesticides Used in Agriculture.

[181] de Jong G. (1991). A study of exposure, health effects and mortality of workers engaged in the manufacture and formulation of the insecticides aldrin and dieldrin. Toxicol. Lett..

[182] Cavender F.L., Cook B.T., Page N.P. (1988). Carcinogenicity Assessment of Aldrin and Dieldrin.

[183] National Academy of Sciences (1982). Committee on Toxicology, an Assessment of the Health Risks of Seven Pesticides Used for Termite Control.

[184] Jorgenson J.L. (2001). Aldrin and dieldrin: A review of research on their production, environmental deposition and fate, bioaccumulation, toxicology, and epidemiology in the United States. Environ. Health Perspect..

[185] Rodan B.D., Pennington D.W., Eckley N., Boethling R.S. (1999). Screening for Persistent Organic Pollutants: Techniques To Provide a Scientific Basis for POPs Criteria in International Negotiations. Environ. Sci. Technol..

[186] ATSDR (2002). Toxicological Profile for Aldrin/Dieldrin.

[187] Zhao X., Salgado V.L., Yeh J.Z., Narahashi T. (2003). Differential Actions of Fipronil and Dieldrin Insecticides on GABA-Gated Chloride Channels in Cockroach Neurons. J. Pharmacol. Exp. Ther..

[188] Fleming L., Mann J.B., Bean J., Briggle T., Sanchez-Ramos J.R. (1994). Parkinson’s disease and brain levels of organochlorine pesticides. Ann. Neurol..

[189] Corrigan F.M., French M., Murray L. (1996). Organochlorine compounds in human brain. Hum. Exp. Toxicol..

[190] Corrigan F., Murray L., Wyatt C., Shore R. (1998). Diorthosubstituted polychlorinated biphenyls in caudate nucleus in Parkinson’s disease. Exp. Neurol..

[191] Sanchez-Ramos J., Facca A., Basit A., Song S. (1998). Toxicity of Dieldrin for Dopaminergic Neurons in Mesencephalic Cultures. Exp. Neurol..

[192] Chhillar N., Singh N.K., Banerjee B.D., Bala K., Mustafa, Sharma D., Chhillar M. (2013). Organochlorine Pesticide Levels and Risk of Parkinson’s Disease in North Indian Population. ISRN Neurol..

[193] Weisskopf M.G., Knekt P., O’Reilly E.J., Lyytinen J., Reunanen A., Laden F., Altshul L., Ascherio A. (2010). Persistent organochlorine pesticides in serum and risk of Parkinson disease. Neurology.

[194] Richardson J.R., Caudle W.M., Wang M., Dean E.D., Pennell K.D., Miller G.W. (2006). Developmental exposure to the pesticide dieldrin alters the dopamine system and increases neurotoxicity in an animal model of Parkinson’s disease. Faseb. J..

[195] Gezer A.O., Kochmanski J., VanOeveren S.E., Cole-Strauss A., Kemp C.J., Patterson J.R., Miller K.M., Kuhn N.C., Herman D.E., McIntire A. (2020). Developmental exposure to the organochlorine pesticide dieldrin causes male-specific exacerbation of α-synuclein-preformed fibril-induced toxicity and motor deficits. Neurobiol. Dis..

[196] Kitazawa M., Anantharam V., Kanthasamy A.G. (2001). Dieldrin-induced oxidative stress and neurochemical changes contribute to apoptopic cell death in dopaminergic cells. Free Radic. Biol. Med..

[197] Kitazawa M., Anantharam V., Kanthasamy A., Kanthasamy A.G. (2004). Dieldrin Promotes Proteolytic Cleavage of Poly(ADP-Ribose) Polymerase and Apoptosis in Dopaminergic Cells: Protective Effect of Mitochondrial Anti-Apoptotic Protein Bcl-2. NeuroToxicology.

[198] Sharma H., Zhang P., Barber D.S., Liu B. (2010). Organochlorine pesticides dieldrin and lindane induce cooperative toxicity in dopaminergic neurons: Role of oxidative stress. NeuroToxicology.

[199] Schmidt J.T., Rushin A., Boyda J., Souders C.L., Martyniuk C.J. (2017). Dieldrin-induced neurotoxicity involves impaired mitochondrial bioenergetics and an endoplasmic reticulum stress response in rat dopaminergic cells. NeuroToxicology.

[200] Kochmanski J., VanOeveren S.E., Patterson J.R., Bernstein A. (2019). Developmental Dieldrin Exposure Alters DNA Methylation at Genes Related to Dopaminergic Neuron Development and Parkinson’s Disease in Mouse Midbrain. Toxicol. Sci..

[201] Horning K.J., Caito S.W., Tipps K.G., Bowman A.B., Aschner M. (2015). Manganese Is Essential for Neuronal Health. Annu. Rev. Nutr..

[202] Greger J.L. (1999). Nutrition versus toxicology of manganese in humans: Evaluation of potential biomarkers. NeuroToxicology.

[203] Blanc P.D. (2018). The early history of manganese and the recognition of its neurotoxicity, 1837–1936. NeuroToxicology.

[204] O’Neal S.L., Zheng W. (2015). Manganese Toxicity upon Overexposure: A Decade in Review. Curr. Environ. Health Rep..

[205] Tuschl K., Mills P.B., Clayton P.T. (2013). Manganese and the Brain. Int. Rev. Neurobiol..

[206] Walter E., Alsaffar S., Livingstone C., Ashley S.L. (2016). Manganese toxicity in critical care: Case report, literature review and recommendations for practice. J. Intensiv. Care Soc..

[207] Tanner C.M., Ross G.W., Jewell S.A., Hauser R.A., Jankovic J., Factor S.A., Bressman S., Deligtisch A., Marras C., Lyons K.E. (2009). Occupation and risk of parkinsonism: A multicenter case-control study. Arch. Neurol..

[208] Pal P.K., Samii A., Calne D.B. (1999). Manganese neurotoxicity: A review of clinical features, imaging and pathology. NeuroToxicology.

[209] Zota A.R., Riederer A.M., Ettinger A.S., Schaider L.A., Shine J.P., Amarasiriwardena C.J., Wright R., Spengler J.D. (2015). Associations between metals in residential environmental media and exposure biomarkers over time in infants living near a mining-impacted site. J. Expo. Sci. Environ. Epidemiol..

[210] Zota A.R., Schaider L.A., Ettinger A.S., Wright R.O., Shine J.P., Spengler J.D. (2011). Metal sources and exposures in the homes of young children living near a mining-impacted Superfund site. J. Expo. Sci. Environ. Epidemiol..

[211] Menezes-Filho J.A., Novaes C.D.O., Moreira J.C., Sarcinelli P.N., Mergler D. (2011). Elevated manganese and cognitive performance in school-aged children and their mothers. Environ. Res..

[212] Haynes E.N., Sucharew H., Hilbert T.J., Kuhnell P., Spencer A., Newman N.C., Burns R., Wright R., Parsons P.J., Dietrich K.N. (2017). Impact of air manganese on child neurodevelopment in East Liverpool, Ohio. NeuroToxicology.

[213] Flynn M.R., Susi P. (2009). Neurological risks associated with manganese exposure from welding operations—A literature review. Int. J. Hyg. Environ. Health.

[214] Stepens A., Logina I., Liguts V., Aldiņš P., Ekšteina I., Platkājis A., Mārtiņsone I., Tērauds E., Rozentāle B., Donaghy M. (2008). A Parkinsonian Syndrome in Methcathinone Users and the Role of Manganese. N. Engl. J. Med..

[215] Varlibas F., Delipoyraz I., Yuksel G., Filiz G., Tireli H., Gecim N.O. (2009). Neurotoxicity following chronic intravenous use of “Russian cocktail”. Clin. Toxicol..

[216] Crossgrove J., Zheng W. (2004). Manganese toxicity upon overexposure. NMR Biomed..

[217] Lucchini R., Bergamaschi E., Smargiassi A., Festa D., Apostoli P. (1997). Motor Function, Olfactory Threshold, and Hematological Indices in Manganese-Exposed Ferroalloy Workers. Environ. Res..

[218] Meyer-Baron M., Schäper M., Knapp G., Lucchini R., Zoni S., Bast-Pettersen R., Ellingsen D.G., Thomassen Y., He S., Yuan H. (2013). The neurobehavioral impact of manganese: Results and challenges obtained by a meta-analysis of individual participant data. NeuroToxicology.

[219] Roth J.A., Garrick M. (2003). Iron interactions and other biological reactions mediating the physiological and toxic actions of manganese. Biochem. Pharmacol..

[220] Peres T.V., Eyng H., Lopes S.C., Colle D., Gonçalves F.M., Venske D.K.R., Lopes M.W., Ben J., Bornhorst J., Schwerdtle T. (2015). Developmental exposure to manganese induces lasting motor and cognitive impairment in rats. NeuroToxicology.

[221] Sanyal J., Ahmed S.S.S.J., Ng H.K.T., Naiya T., Ghosh E., Banerjee T.K., Lakshmi J., Guha G., Rao V.R. (2016). Metallomic Biomarkers in Cerebrospinal fluid and Serum in patients with Parkinson’s disease in Indian population. Sci. Rep..

[222] Fukushima T., Tan X., Luo Y., Kanda H. (2009). Relationship between Blood Levels of Heavy Metals and Parkinson’s Disease in China. Neuroepidemiology.

[223] Brenneman K.A., Wong B.A., Buccellato M.A., Costa E.R., Gross E.A., Dorman D.C. (2000). Direct Olfactory Transport of Inhaled Manganese (54MnCl2) to the Rat Brain: Toxicokinetic Investigations in a Unilateral Nasal Occlusion Model. Toxicol. Appl. Pharmacol..

[224] Frisbie S.H., Mitchell E.J., Roudeau S., Domart F., Carmona A., Ortega R. (2019). Manganese levels in infant formula and young child nutritional beverages in the United States and France: Comparison to breast milk and regulations. PLoS ONE.

[225] Chen P., Bornhorst J., Aschner M. (2018). Manganese metabolism in humans. Front Biosci..

[226] Liu C., Jursa T., Aschner M., Smith D.R., Mukhopadhyay S. (2021). Up-regulation of the manganese transporter SLC30A10 by hypoxia-inducible factors defines a homeostatic response to manganese toxicity. Proc. Natl. Acad. Sci. USA.

[227] Omokhodion F.O., Howard J.M. (1994). Trace elements in the sweat of acclimatized persons. Clin. Chim. Acta.

[228] O’Neal S.L., Hong L., Fu S., Jiang W., Jones A., Nie L.H., Zheng W. (2014). Manganese accumulation in bone following chronic exposure in rats: Steady-state concentration and half-life in bone. Toxicol. Lett..

[229] Dierschke K., Isaxon C., Andersson U.B.K., Assarsson E., Axmon A., Stockfelt L., Gudmundsson A., Jönsson B.A.G., Kåredal M., Londahl J. (2017). Acute respiratory effects and biomarkers of inflammation due to welding-derived nanoparticle aggregates. Int. Arch. Occup. Environ. Health.

[230] Tarale P., Chakrabarti T., Sivanesan S., Naoghare P., Bafana A., Krishnamurthi K. (2016). Potential Role of Epigenetic Mechanism in Manganese Induced Neurotoxicity. BioMed Res. Int..

[231] Cantone L., Nordio F., Hou L., Apostoli P., Bonzini M., Tarantini L., Angelici L., Bollati V., Zanobetti A., Schwartz J. (2011). Inhalable Metal-Rich Air Particles and Histone H3K4 Dimethylation and H3K9 Acetylation in a Cross-sectional Study of Steel Workers. Environ. Health Perspect..

[232] Bollati V., Marinelli B., Apostoli P., Bonzini M., Nordio F., Hoxha M., Pegoraro V., Motta V., Tarantini L., Cantone L. (2010). Exposure to Metal-Rich Particulate Matter Modifies the Expression of Candidate MicroRNAs in Peripheral Blood Leukocytes. Environ. Health Perspect..

[233] Harischandra D.S., Ghaisas S., Rokad D., Zamanian M., Jin H., Anantharam V., Kimber M., Kanthasamy A., Kanthasamy A.G. (2018). Environmental neurotoxicant manganese regulates exosome-mediated extracellular miRNAs in cell culture model of Parkinson’s disease: Relevance to α-synuclein misfolding in metal neurotoxicity. Neurotoxicology.

[234] Castillo S., Muñoz P., Behrens M.I., Diaz-Grez F., Segura-Aguilar J. (2017). On the Role of Mining Exposure in Epigenetic Effects in Parkinson’s Disease. Neurotox. Res..

[235] Nwanaji-Enwerem J.C., Colicino E., Specht A.J., Gao X., Wang C., Vokonas P., Weisskopf M.G., Boyer E.W., Baccarelli A.A., Schwartz J. (2020). Individual species and cumulative mixture relationships of 24-hour urine metal concentrations with DNA methylation age variables in older men. Environ. Res..

[236] Fu S., O’Neal S., Hong L., Jiang W., Zheng W. (2015). Elevated Adult Neurogenesis in Brain Subventricular Zone Following In vivo Manganese Exposure: Roles of Copper and DMT1. Toxicol. Sci..

[237] Fu S., Jiang W., Gao X., Zeng A., Cholger D., Cannon J., Chen J., Zheng W. (2016). Aberrant Adult Neurogenesis in the Subventricular Zone-Rostral Migratory Stream-Olfactory Bulb System Following Subchronic Manganese Exposure. Toxicol. Sci..

[238] Horgusluoglu E., Nudelman K., Nho K., Saykin A.J. (2016). Adult neurogenesis and neurodegenerative diseases: A systems biology perspective. Am. J. Med Genet. Part B Neuropsychiatr. Genet..

[239] Lim D.A., Alvarez-Buylla A. (2016). The Adult Ventricular–Subventricular Zone (V-SVZ) and Olfactory Bulb (OB) Neurogenesis. Cold Spring Harb. Perspect. Biol..

[240] Marxreiter F., Regensburger M., Winkler J. (2012). Adult neurogenesis in Parkinson’s disease. Cell Mol. Life Sci..

[241] Ng K.L., Li J.-D., Cheng M.Y., Leslie F.M., Lee A.G., Zhou Q.-Y. (2005). Dependence of Olfactory Bulb Neurogenesis on Prokineticin 2 Signaling. Science.

[242] Wang L., Ohishi T., Shiraki A., Morita R., Akane H., Ikarashi Y., Mitsumori K., Shibutani M. (2012). Developmental Exposure to Manganese Chloride Induces Sustained Aberration of Neurogenesis in the Hippocampal Dentate Gyrus of Mice. Toxicol. Sci..

[243] Cheng M., Leslie F.M., Zhou Q.-Y. (2006). Expression of prokineticins and their receptors in the adult mouse brain. J. Comp. Neurol..

[244] Zoni S., Bonetti G., Lucchini R. (2012). Olfactory functions at the intersection between environmental exposure to manganese and Parkinsonism. J. Trace Elem. Med. Biol. Organ Soc. Miner. Trace Elem. (GMS).

[245] Mogi M., Harada M., Kondo T., Riederer P., Inagaki H., Minami M., Nagatsu T. (1994). Interleukin-1 beta, interleukin-6, epidermal growth factor and transforming growth factor-alpha are elevated in the brain from parkinsonian patients. Neurosci. Lett..

[246] Freeman L.C., Ting J.P.-Y. (2015). The pathogenic role of the inflammasome in neurodegenerative diseases. J. Neurochem..

[247] Pajarillo E., Johnson J., Rizor A., Nyarko-Danquah I., Adinew G., Bornhorst J., Stiboller M., Schwerdtle T., Son D.-S., Aschner M. (2020). Astrocyte-specific deletion of the transcription factor Yin Yang 1 in murine substantia nigra mitigates manganese-induced dopaminergic neurotoxicity. J. Biol. Chem..

[248] Huang Y., Wen Q., Huang J., Luo M., Xiao Y., Mo R., Wang J. (2021). Manganese (II) chloride leads to dopaminergic neurotoxicity by promoting mitophagy through BNIP3-mediated oxidative stress in SH-SY5Y cells. Cell. Mol. Biol. Lett..

[249] Zhang D., Kanthasamy A., Anantharam V., Kanthasamy A. (2011). Effects of manganese on tyrosine hydroxylase (TH) activity and TH-phosphorylation in a dopaminergic neural cell line. Toxicol. Appl. Pharmacol..

[250] Ma Z., Liu K., Li X.R., Wang C., Liu C., Yan D.Y., Deng Y., Liu W., Xu B. (2020). Alpha-synuclein is involved in manganese-induced spatial memory and synaptic plasticity impairments via TrkB/Akt/Fyn-mediated phosphorylation of NMDA receptors. Cell Death Dis..

[251] Choi C.J., Anantharam V., Martin D.P., Nicholson E.M., Richt J.A., Kanthasamy A., Kanthasamy A.G. (2010). Manganese Upregulates Cellular Prion Protein and Contributes to Altered Stabilization and Proteolysis: Relevance to Role of Metals in Pathogenesis of Prion Disease. Toxicol. Sci..

[252] Ohishi T., Wang L., Akane H., Shiraki A., Goto K., Ikarashi Y., Suzuki K., Mitsumori K., Shibutani M. (2012). Reversible aberration of neurogenesis affecting late-stage differentiation in the hippocampal dentate gyrus of rat offspring after maternal exposure to manganese chloride. Reprod. Toxicol..

[253] Haikal C., Chen Q.Q., Li J.Y. (2019). Microbiome changes: An indicator of Parkinson’s disease?. Transl. Neurodegener..

[254] Yang D., Zhao D., Ali Shah S.Z., Wu W., Lai M., Zhang X., Li J., Guan Z., Zhao H., Li W. (2019). The Role of the Gut Microbiota in the Pathogenesis of Parkinson’s Disease. Front. Neurol..

[255] Koller W.C., Lyons K.E., Truly W. (2004). Effect of levodopa treatment for parkinsonism in welders: A double-blind study. Neurology.

[256] Jiang Y.-M., Mo X.-A., Du F.-Q., Fu X., Zhu X.-Y., Gao H.-Y., Xie J.-L., Liao F.-L., Pira E., Zheng W. (2006). Effective Treatment of Manganese-Induced Occupational Parkinsonism with p-Aminosalicylic Acid: A Case of 17-Year Follow-Up Study. J. Occup. Environ. Med..

[257] Zheng W., Jiang Y.-M., Zhang Y., Jiang W., Wang X., Cowan D.M. (2009). Chelation therapy of manganese intoxication with para-aminosalicylic acid (PAS) in Sprague–Dawley rats. NeuroToxicology.

[258] Ky S.Q., Deng H.S., Xie P.Y., Hu W. (1992). A report of two cases of chronic serious manganese poisoning treated with sodium para-aminosalicylic acid. Occup. Environ. Med..

[259] Ahmadi N., Ghanbarinejad V., Ommati M.M., Jamshidzadeh A., Heidari R. (2018). Taurine prevents mitochondrial membrane permeabilization and swelling upon interaction with manganese: Implication in the treatment of cirrhosis-associated central nervous system complications. J. Biochem. Mol. Toxicol..

[260] Ommati M.M., Heidari R., Ghanbarinejad V., Abdoli N., Niknahad H. (2018). Taurine Treatment Provides Neuroprotection in a Mouse Model of Manganism. Biol. Trace Elem. Res..

[261] Lu C.-L., Tang S., Meng Z.-J., He Y.-Y., Song L.-Y., Liu Y.-P., Ma N., Li X.-Y., Guo S.-C. (2014). Taurine improves the spatial learning and memory ability impaired by sub-chronic manganese exposure. J. Biomed. Sci..

[262] Gitler A.D., Chesi A., Geddie M.L., Strathearn K.E., Hamamichi S., Hill K.J., Caldwell K.A., Caldwell G.A., Cooper A.A., Rochet J.C. (2009). Alpha-Synuclein is part of a diverse and highly conserved interaction network that includes PARK9 and manganese toxicity. Nat. Genet..

[263] Tan J., Zhang T., Jiang L., Chi J., Hu D., Pan Q., Wang D., Zhang Z. (2011). Regulation of Intracellular Manganese Homeostasis by Kufor-Rakeb Syndrome-associated ATP13A2 Protein. J. Biol. Chem..

[264] Harischandra D.S., Jin H., Anantharam V., Kanthasamy A., Kanthasamy A.G. (2014). α-Synuclein Protects Against Manganese Neurotoxic Insult During the Early Stages of Exposure in a Dopaminergic Cell Model of Parkinson’s Disease. Toxicol. Sci..

[265] Pessoa J.C., Etcheverry S., Gambino D. (2015). Vanadium compounds in medicine. Coord. Chem. Rev..

[266] Rehder D. (2008). Bioinorganic Vanadium Chemistry.

[267] Rehder D. (2012). The potentiality of vanadium in medicinal applications. Future Med. Chem..

[268] Barceloux D.G. (1999). Vanadium. J. Toxicol. Clin. Toxicol..

[269] Amorim F.A.C., Welz B., Costa A.C.S., Lepri F.G., Vale M.G.R., Ferreira S.L.C. (2007). Determination of vanadium in petroleum and petroleum products using atomic spectrometric techniques. Talanta.

[270] Pyrzyńska K., Wierzbicki T. (2004). Determination of vanadium species in environmental samples. Talanta.

[271] McNeilly J.D., Heal M.R., Beverland I.J., Howe A., Gibson M.D., Hibbs L.R., MacNee W., Donaldson K. (2004). Soluble transition metals cause the pro-inflammatory effects of welding fumes in vitro. Toxicol. Appl. Pharmacol..

[272] Schlesinger W.H., Klein E.M., Vengosh A. (2017). Global biogeochemical cycle of vanadium. Proc. Natl. Acad. Sci. USA.

[273] Ścibior A., Pietrzyk ., Plewa Z., Skiba A. (2020). Vanadium: Risks and possible benefits in the light of a comprehensive overview of its pharmacotoxicological mechanisms and multi-applications with a summary of further research trends. J. Trace Elem. Med. Biol..

[274] Thompson K.H., Lichter J., LeBel C., Scaife M.C., McNeill J.H., Orvig C. (2009). Vanadium treatment of type 2 diabetes: A view to the future. J. Inorg. Biochem..

[275] Korbecki J., Baranowska-Bosiacka I., Gutowska I., Chlubek D. (2012). Biochemical and medical importance of vanadium compounds. Acta Biochim. Pol..

[276] Shechter Y., Shisheva A. (1993). Vanadium salts and the future treatment of diabetes. Endeavour.

[277] Bishayee A., Waghray A., Patel M.A., Chatterjee M. (2010). Vanadium in the detection, prevention and treatment of cancer: The in vivo evidence. Cancer Lett..

[278] Myron D.R., Givand S.H., Nielsen F.H. (1977). Vanadium content of selected foods as determined by flameless atomic absorption spectroscopy. J. Agric. Food Chem..

[279] Byrne A., Kosta L. (1978). Vanadium in foods and in human body fluids and tissues. Sci. Total Environ..

[280] Hansen T.V., Aaseth J., Alexander J. (1982). The effect of chelating agents on vanadium distribution in the rat body and on uptake by human erythrocytes. Arch. Toxicol..

[281] Mussali-Galante P., Rodríguez-Lara V., Hernández-Tellez B., Avila-Costa M.R., Colín-Barenque L., Bizarro-Nevarez P., Martínez-Levy G., Rojas-Lemus M., Piñón-Zarate G., Saldivar-Osorio L. (2005). Inhaled vanadium pentoxide decrease gamma-tubulin of mouse testes at different exposure times. Toxicol. Ind. Health.

[282] Kiss T., Kiss E., Garribba E., Sakurai H. (2000). Speciation of insulin-mimetic VO(IV)-containing drugs in blood serum. J. Inorg. Biochem..

[283] Fatola O.I., Olaolorun F.A., Olopade F.E., Olopade J.O. (2019). Trends in vanadium neurotoxicity. Brain Res. Bull..

[284] Garcia G.B., Biancardi M.E., Quiroga A.D. (2005). Vanadium (V)-Induced Neurotoxicity in the Rat Central Nervous System: A Histo-Immunohistochemical Study. Drug Chem. Toxicol..

[285] Sharma R.P., Coulombe R.A., Srisuchart B. (1986). Effects of dietary vanadium exposure on levels of regional brain neurotransmitters and their metabolites. Biochem. Pharmacol..

[286] Sanchez D.J., Colomina M.T., Domingo J.L. (1998). Effects of Vanadium on Activity and Learning in Rats. Physiol. Behav..

[287] Folarin O., Olopade F., Onwuka S., Olopade J. (2016). Memory Deficit Recovery after Chronic Vanadium Exposure in Mice. Oxidative Med. Cell. Longev..

[288] Soazo M., Garcia G.B. (2007). Vanadium exposure through lactation produces behavioral alterations and CNS myelin deficit in neonatal rats. Neurotoxicol. Teratol..

[289] Mustapha O.A., Oke B., Offen N., Sirén A.-L., Olopade J. (2014). Neurobehavioral and cytotoxic effects of vanadium during oligodendrocyte maturation: A protective role for erythropoietin. Environ. Toxicol. Pharmacol..

[290] Ohiomokhare S., Olaolorun F., Ladagu A., Olopade F., Howes M.-J., Okello E., Olopade J., Chazot P. (2020). The Pathopharmacological Interplay between Vanadium and Iron in Parkinson’s Disease Models. Int. J. Mol. Sci..

[291] Zhou D.-L., Feng C.-Y., Lan Y.-J., Wang Z.-M., Huang S., Wang M.-Z., Zhu T. (2007). Paired-control study on the effect of vanadium on neurobehavioral functions. Sichuan Da Xue Xue Bao. Yi Xue Ban J. Sichuan Univ. Med Sci. Ed..

[292] Li H., Zhou D., Zhang Q., Feng C., Zheng W., He K., Lan Y. (2013). Vanadium exposure-induced neurobehavioral alterations among Chinese workers. NeuroToxicology.

[293] Schlake H.-P., Bertram H.P., Husstedt I.W., Schuierer G. (1994). Acute systemic vanadate poisoning presenting as cerebrovascular ischemia with prolonged reversible neurological deficits (PRIND). Clin. Neurol. Neurosurg..

[294] Bonner J.C., Rice A.B., Moomaw C.R., Morgan D.L. (2000). Airway fibrosis in rats induced by vanadium pentoxide. Am. J. Physiol. Cell. Mol. Physiol..

[295] Woodin M.A., Liu Y., Neuberg D., Hauser R., Smith T.J., Christiani D.C. (2000). Acute respiratory symptoms in workers exposed to vanadium-rich fuel-oil ash. Am. J. Ind. Med..

[296] Irsigler G.B., Visser P.J., Spangenberg P.A. (1999). Asthma and chemical bronchitis in vanadium plant workers. Am. J. Ind. Med..

[297] Fortoul T.I., Piñón-Zárate G., Diaz-Bech M.E., Gonzalez-Villalva A., Mussali-Galante P., Rodriguez-Lara V., Colin-Barenque L., Martinez-Pedraza M., Montaño L.F. (2008). Spleen and bone marrow megakaryocytes as targets for inhaled vanadium. Histol. Histopathol..

[298] González-Villalva A.E., Falcon-Rodriguez C.I., Der Goes T.I.F.-V. (2010). Signaling pathways involved in megakaryopoiesis. Gac. Med. Mex.

[299] Gonzalez-Villalva A., Fortoul T.I., Avila-Costa M.R., Piñón-Zárate G., Rodriguez-Laraa V., Martínez-Levy G.A., Rojas-Lemus M., Bizarro-Nevarez P., Díaz-Bech P., Mussali-Galante P. (2006). Thrombocytosis induced in mice after subacute and subchronic V2O5 inhalation. Toxicol. Ind. Health.

[300] Al-Bayati M.A., Giri S.N., Raabe O.G., Rosenblatt L.S., Shifrine M. (1989). Time and dose-response study of the effects of vanadate on rats: Morphological and biochemical changes in organs. J. Environ. Pathol. Toxicol. Oncol..

[301] Lahav M., Rennert H., Barzilai D. (1986). Inhibition by vanadate of cyclic AMP production in rat corpora lutea incubated in vitro. Life Sci..

[302] Fortoul T., Bizarronevares P., Acevedonava S., Pinonzarate G., Rodriguezlara V., Colinbarenque L., Mussaligalante P., Avilacasado M., Avilacosta M., Saldivarosorio L. (2007). Ultrastructural findings in murine seminiferous tubules as a consequence of subchronic vanadium pentoxide inhalation. Reprod. Toxicol..

[303] Sjoberg S.G. (1950). Vanadium pentoxide Dust. A clinical and experimental investigation on its effect after inhalation. Acta Med. Scand. Suppl..

[304] Valko M., Morris H., Cronin M.T. (2005). Metals, toxicity and oxidative stress. Curr. Med. Chem..

[305] Todorich B., Olopade J.O., Surguladze N., Zhang X., Neely E., Connor J.R. (2010). The Mechanism of Vanadium-Mediated Developmental Hypomyelination Is Related to Destruction of Oligodendrocyte Progenitors Through a Relationship with Ferritin and Iron. Neurotox. Res..

[306] Ding M., Gannett P., Rojanasakul Y., Liu K., Shi X. (1994). One-electron reduction of vanadate by ascorbate and related free radical generation at physiological pH. J. Inorg. Biochem..

[307] Shi X., Dalal N.S. (1992). Hydroxyl Radical Generation in the Nadh/Microsomal Reduction of vanadate. Free Radic. Res. Commun..

[308] Shi X., Dalal N. (1993). Vanadate-Mediated Hydroxyl Radical Generation from Superoxide Radical in the Presence of NADH: Haber-Weiss vs Fenton Mechanism. Arch. Biochem. Biophys..

[309] Capella L.S., Gefé M.R., Silva E.F., Affonso-Mitidieri O., Lopes A.G., Rumjanek V.M., Capella M.A. (2002). Mechanisms of vanadate-induced cellular toxicity: Role of cellular glutathione and NADPH. Arch. Biochem. Biophys..

[310] Crans D.C., Zhang B., Gaidamauskas E., Keramidas A.D., Willsky G.R., Roberts C.R. (2010). Is vanadate reduced by thiols under biological conditions? Changing the redox potential of V(V)/V(IV) by complexation in aqueous solution. Inorg. Chem..

[311] Huyer G., Liu S., Kelly J., Moffat J., Payette P., Kennedy B., Tsaprailis G., Gresser M.J., Ramachandran C. (1997). Mechanism of Inhibition of Protein-tyrosine Phosphatases by Vanadate and Pervanadate. J. Biol. Chem..

[312] Meng F.-G., Zhang Z.-Y. (2012). Redox regulation of protein tyrosine phosphatase activity by hydroxyl radical. Biochim. Biophys. Acta (BBA) Proteins Proteom..

[313] Sturla L.-M., Amorino G., Alexander M.S., Mikkelsen R.B., Valerie K., Schmidt-Ullrichr R.K. (2005). Requirement of Tyr-992 and Tyr-1173 in Phosphorylation of the Epidermal Growth Factor Receptor by Ionizing Radiation and Modulation by SHP2. J. Biol. Chem..

[314] Lee K., Esselman W.J. (2002). Inhibition of PTPs by H2O2 regulates the activation of distinct MAPK pathways. Free Radic. Biol. Med..

[315] Zhao Z., Tan Z., Diltz C.D., You M., Fischer E.H., Zaia J., Boynton R.E., McIntosh A., Marshak D.R., Olsson H. (1996). Activation of Mitogen-activated Protein (MAP) Kinase Pathway by Pervanadate, a Potent Inhibitor of Tyrosine Phosphatases. J. Biol. Chem..

[316] Chien P.-S., Mak O.-T., Huang H.-J. (2006). Induction of COX-2 protein expression by vanadate in A549 human lung carcinoma cell line through EGF receptor and p38 MAPK-mediated pathway. Biochem. Biophys. Res. Commun..

[317] Migliore L., Coppedè F. (2009). Environmental-induced oxidative stress in neurodegenerative disorders and aging. Mutat. Res. Toxicol. Environ. Mutagen..

[318] Brown T.P., Rumsby P.C., Capleton A.C., Rushton L., Levy L.S. (2006). Pesticides and Parkinson’s disease--is there a link?. Environ. Health Perspect..

[319] Suntres Z.E. (2002). Role of antioxidants in paraquat toxicity. Toxicology.

[320] Uversky V.N. (2004). Neurotoxicant-induced animal models of Parkinson’s disease: Understanding the role of rotenone, maneb and paraquat in neurodegeneration. Cell Tissue Res..

[321] Costa C., Teodoro M., Rugolo C.A., Alibrando C., Giambò F., Briguglio G., Fenga C. (2020). MicroRNAs alteration as early biomarkers for cancer and neurodegenerative diseases: New challenges in pesticides exposure. Toxicol. Rep..

[322] Aloizou A.M., Siokas V., Sapouni E.M., Sita N., Liampas I., Brotis A.G., Rakitskii V.N., Burykina T.I., Aschner M., Bogdanos D.P. (2020). Parkinson’s disease and pesticides: Are microRNAs the missing link?. Sci. Total Environ..

[323] Caggiu E., Paulus K., Mameli G., Arru G., Sechi G.P., Sechi L.A. (2018). Differential expression of miRNA 155 and miRNA 146a in Parkinson’s disease patients. eNeurologicalSci.

[324] Goh S.Y., Chao Y.X., Dheen S.T., Tan E.-K., Tay S.S.-W. (2019). Role of MicroRNAs in Parkinson’s Disease. Int. J. Mol. Sci..

